# Novel Genes Required for Surface-Associated Motility in *Acinetobacter baumannii*

**DOI:** 10.1007/s00284-021-02407-x

**Published:** 2021-03-05

**Authors:** Ulrike Blaschke, Evelyn Skiebe, Gottfried Wilharm

**Affiliations:** Robert Koch Institute, Project group P2, Burgstr. 37, 38855 Wernigerode, Germany

## Abstract

*Acinetobacter baumannii* is an opportunistic and increasingly multi-drug resistant human pathogen rated as a critical priority one pathogen for the development of new antibiotics by the WHO in 2017. Despite the lack of flagella, *A. baumannii* can move along wet surfaces in two different ways: via twitching motility and surface-associated motility. While twitching motility is known to depend on type IV pili, the mechanism of surface-associated motility is poorly understood. In this study, we established a library of 30 *A. baumannii* ATCC® 17978™ mutants that displayed deficiency in surface-associated motility. By making use of natural competence, we also introduced these mutations into strain 29D2 to differentiate strain-specific versus species-specific effects of mutations. Mutated genes were associated with purine/pyrimidine/folate biosynthesis (e.g. *purH*, *purF*, *purM*, *purE*), alarmone/stress metabolism (e.g. Ap4A hydrolase), RNA modification/regulation (e.g. methionyl-tRNA synthetase), outer membrane proteins (e.g. *ompA*), and genes involved in natural competence (*comEC*). All tested mutants originally identified as motility-deficient in strain ATCC® 17978™ also displayed a motility-deficient phenotype in 29D2. By contrast, further comparative characterization of the mutant sets of both strains regarding pellicle biofilm formation, antibiotic resistance, and virulence in the *Galleria mellonella* infection model revealed numerous strain-specific mutant phenotypes. Our studies highlight the need for comparative analyses to characterize gene functions in *A*. *baumannii* and for further studies on the mechanisms underlying surface-associated motility.

**Supplementary Information:**

The online version contains supplementary material available at 10.1007/s00284-021-02407-x.

## Introduction

*Acinetobacter baumannii* is a Gram-negative aerobic coccobacillus [1, 2]. Being an opportunistic human pathogen [3], *A. baumannii* is associated with nosocomial diseases including soft tissue, bloodstream, and urinary tract infections as well as pneumonia [2]. Worldwide, about 9% of culture-positive infections found in intensive care units arise from *Acinetobacter *spp*.* [4]. Increased multi-drug resistance in *A.* *baumannii* has become problematic in recent years [5–7]. As a consequence of rising multi-drug resistance, *A. baumannii* was rated as critical priority one pathogen for the development of new antibiotics by the WHO in 2017 [8]. Drug resistance and environmental persistence have enabled *A. baumannii* to successfully establish in the hospital environment. Some clinical isolates can survive 100 days or more under dry conditions [9–13]. An important factor for the interaction of *A. baumannii* with biotic or abiotic surfaces is the formation of biofilms, a feature that is associated with an increased tolerance to desiccation stress [14].

A connection between *A.* *baumannii* virulence and motility has been shown in the *Caenorhabditis elegans* infection model where hypermotility resulted in increased virulence [15]. Although *A. baumannii* does not produce flagella, it is capable of moving in two different ways: via twitching motility and surface-associated motility. For *A.* *baumannii*, twitching motility has been shown to depend on type IV pili (T4P) [16, 17] which drive the bacteria via retraction of attached T4P [18–25]. Inactivation of the putative T4P retraction ATPase *pilT* reduces twitching motility [11, 26–28] but does not abolish surface-associated motility [16, 26]. Surface-associated motility in *A.* *baumannii* occurs at the surface of semi-dry media and is independent of T4P [26, 29]. Surface-associated motility is poorly understood mechanistically, but was demonstrated to be controlled by quorum sensing [26], light [30], iron availability [31, 32], and to depend on the surfactant-like compound acinetin 505 [26, 33, 34]. Also, synthesis of 1,3-diaminopropane (DAP) [35] and lipopolysaccharide (LPS) [32] were shown to contribute to surface-associated motility of *A.* *baumannii*. Several additional genes have been identified which contribute to *A. baumannii*’s capacity for surface-associated motility [26, 32, 36], including a ribonuclease T2 family protein [37] and superoxide dismutase [38]. A recent study revealed the regulatory control of surface-associated motility and biofilm formation by a cyclic-di-GMP signaling network in *A. baumannii* strain ATCC 17978 [39]. Interestingly, studies on phase-variable phenotypes in *A. baumannii* strain AB5075 showed that “opaque phase” bacterial colonies had improved surface-associated motility [40, 41]. A correlation between pellicle biofilm formation and surface-associated motility has been described in *A. baumannii* [42]. Given the fact that many *A. baumannii* clinical isolates exhibit surface-associated motility, it could be an important trait associated with infection [26, 28, 35].

To investigate the mechanisms underlying surface-associated motility, we utilized a previously generated transposon mutant library of ATCC® 17978™ [35] which we screened for a surface-associated motility-deficient phenotype. The motility-deficient mutations were found to affect purine/pyrimidine/folate biosynthesis, alarmone/stress metabolism, RNA modification/regulation, outer membrane proteins, and DNA modification. We characterized these mutants with respect to growth, pellicle biofilm formation, antibiotic resistance, and virulence in the *Galleria mellonella* infection model. To facilitate distinguishing between strain-specific and species-specific traits some mutations were also introduced into the naturally competent *A. baumannii* strain 29D2 [43].

## Materials and Methods

### Bacterial Strains and Culture Conditions

*Acinetobacter baumannii* strain ATCC® 17978™ (abbrev. ATCC 17978 subsequently) was purchased from LGC Promochem. The *A. baumannii* strain 29D2 was isolated from a white stork [43] and is naturally competent [44]. All strains were grown at 37 °C in Luria–Bertani (LB) broth or on LB agar, and mutants were supplemented with 50 µg/mL of kanamycin. All strains used in this work are listed in Supplementary Table S1. Single colonies (approx. 10^6^ CFU) were used as inoculum for overnight cultures or motility plates. Neither strain ATCC 17978 nor strain 29D2 exhibited phase variation [40, 41, 45].

### Bacterial Transformation and Generation of an *A. baumannii* Mutant Library

*Acinetobacter baumannii* ATCC 17978 transposon mutants were generated using the EZ-Tn5™ < KAN-2 > Insertion Kit (Epicentre Biotechnologies) as previously described [35]. Transformation of the transposome complex into ATCC 17978 was performed by electroporation [46]. *A. baumannii* 29D2 mutants were generated by making use of the strain’s ability for natural competence. The transforming DNA was isolated from the ATCC 17978 mutants described above. A suspension of DNA-accepting bacteria was generated by resuspending a few colonies (approx. 5 × 10^6^ CFU) in 100 µL of sterile phosphate buffered saline (PBS). The bacterial suspension was then mixed with equal volumes of the transforming DNA (~ 400 ng/µL). This mixture was stabbed into motility agar plates ten times, pipetting 2 µL of the mixture with each stabbing [16]. Controls were wildtype DNA and TE buffer without DNA that were mixed with bacterial suspensions and processed accordingly. The motility plates were incubated for 18 h at 37 °C. After incubation, the bacteria were flushed off the motility plates with 1 mL of sterile PBS and 100 µL (approx. 2 × 10^8^ CFU) was plated on selective agar plates (50 g/mL of kanamycin). After sub-culturing of selected colonies transformation was confirmed by PCR.

### Identification of Transposon Insertion Sites By Single-Primer PCR

To identify the transposon insertion sites of ATCC 17978 motility mutants, single-primer PCR was performed as described previously [35] using one of the following primers targeting EZ-Tn5™ < KAN-2 > : FP-2Kana 5′-CTTCCCGACAACGCAGACCG-3′; FP-3Kana 5′-GAGTTGAAGGATCAGATCACGC-3′; RP-2Kana 5′-CCCTTGTATTACTGTTTATGTAAGC-3′; RP-3Kana 5′-CGCGGCCTCGAGCAAGACG-3′; Tn5-Kana-For4 5′-GTTTTCTCCTTCATTACAGAAACG-3′; and Tn5-Kana-Rev4 5′-CCCATACAATCGATAGATTGTCG-3′. Transposon insertions of all mutants (ATCC 17978 and 29D2) were confirmed by PCR using primers for the EZ-Tn5™ < KAN-2 > kanamycin cassette (Supplementary Fig. S1), which are specified in the manufacturer’s instructions, and appropriate gene target site primers (Supplementary Table S2, Supplementary Figs. S2, S3).

### Surface-Associated Motility

Motility assays were performed as described previously [35]. A single bacterial colony (approx. 10^6^ CFU) from a nutrient agar plate (Oxoid) or selective agar plates (supplemented with 50 µg/mL of kanamycin for the mutants) of either wildtype (ATCC 17978 and 29D2) or mutants was lifted with a pipette tip and transferred to the surface of a motility plate (0.5% agarose). Plates were incubated for 16 h at 37 °C. The diameter of the surface motility spreading zone was measured and quadruplicates were statistically analyzed.

### Bacterial Growth Curves

Growth curves were determined by growing overnight cultures at 37 °C in LB medium (supplemented with 50 µg/mL of kanamycin for the mutants). Overnight cultures were adjusted to one optical density (OD) measured at a wavelength of 600 nm in LB medium. In 250 mL baffled flasks, 50 mL of LB medium (without antibiotics) was inoculated with 1 mL of the OD-adjusted inoculum. The cultures were incubated at 37 °C for 9 h with shaking at 160 rpm. OD measurements at 600 nm were performed every hour by sampling 100 µL of every culture. For each strain, data obtained from 3 independent cultures grown on the same day were averaged and represented by the mean ± SD.

### Infection in the *Galleria mellonella* Caterpillar

For *G. mellonella* caterpillar infection, bacteria were grown in LB medium overnight at 37 °C (50 µg/mL of kanamycin was added to mutant strains). Cultures were diluted 1:50 in LB medium and incubated for another 4 h at 37 °C. Bacteria were pelleted for 5 min at 10,000×*g* at room temperature (RT) and the supernatant was discarded. Bacteria were resuspended in 500 µL sterile PBS, adjusted to an OD_600_ nm of 1.0 and diluted 1:10 in sterile PBS. 5 µL of this dilution, corresponding to 3 × 10^5^ colony-forming units (CFUs), was injected into the last right proleg of *G. mellonella* caterpillars (purchased from TZ-TERRARISTIK, Germany, and BioSystems Technology TruLarv, UK). As a control, caterpillars were injected with 5 µL of sterile PBS. Three independent experiments were performed with groups of 16 caterpillars for every bacterial strain and control. The caterpillars were incubated at 37 °C for 5 days and checked daily for vitality. Experiments with more than two dead caterpillars within 5 days in the control group were not considered valid. CFUs were determined by serial dilutions, plated on nutrient agar, and colonies were counted after incubation at 37 °C for 18 h. For each strain, data obtained from three independent experiments were averaged and represented by the mean ± SD.

### Determination of Susceptibility to Antibiotics

For the minimal inhibitory concentration (MIC) tests, bacteria were grown in LB medium overnight at 37 °C (to mutant strains 50 µg/mL of kanamycin was added). Cultures were diluted 1:50 in LB medium and incubated (without antibiotics) another 4 h at 37 °C. Agar plates were flushed with 2 mL of each culture (approx. 10^8^ CFU/mL) and E-test strips (Liofilchem, Italy) were deposited on nutrient agar plates. MICs were determined after incubation for 16 h at 37 °C. Three independent experiments were performed and statistical significance was tested by the Student’s *t* test (two-tailed, unpaired).

### Pellicle Biofilm Assays

*Acinetobacter baumannii* strains were grown in LB medium overnight at 37 °C (50 µg/mL of kanamycin was added to mutant strains). The cultures were adjusted to an OD_600_ nm of 1.0 (approx. 2 × 10^8^ CFU/mL) and 3 mL of LB medium (without antibiotics) was inoculated with 15 µL of OD-adjusted culture. Samples were incubated at RT for at least 3 days. The LB medium was removed using a thin cannula and the biofilm (sticking to the tube wall) was stained with a 0.5% crystal violet solution (*w*/*v* in Aqua Bidest) for 20 min. The crystal violet was removed and the biofilm was washed twice with 4 mL Aqua Bidest. The biofilm was scrubbed and flushed off the tube walls with a pipet tip and 96% alcohol solution. The absorption at 550 nm (*A*_550 nm_) was determined. Samples which showed an *A*_550 nm_ > 1.0 were diluted 1:10 with 96% ethanol for measurement. For each strain three independent experiments were performed and statistical significance was analyzed by the Student’s *t* test (two-tailed, unpaired).

### Microscopy

The bacterial strains ATCC 17978, ATCC 17978 *ompA::Km*, 29D2, and 29D2 *ompA::Km* were grown for 16 h at 37 °C under constant shaking. One μL of each bacterial overnight culture was pipetted on a glass slide and analyzed under the bright field microscope (200× magnification).

### Statistical Analysis

All experiments were performed at least three times. Comparison between groups was performed using GraphPad Prism 7 with Student’s *t* test (two-tailed, unpaired). *P* values less than 0.05 were considered to be statistically significant.

## Results

### Surface-Associated Motility

Approximately 2000 transposon mutants of ATCC 17978 were screened for surface-associated motility phenotypes and 30 were identified with motility defects. Previous studies were limited to the characterization of mutations in single strains [17, 26, 30]. Here, to provide a comparative study, we introduced at least one mutation of every gene function category into 29D2 to get insight into strain-specific and species-specific traits.

**Fig. 1:**
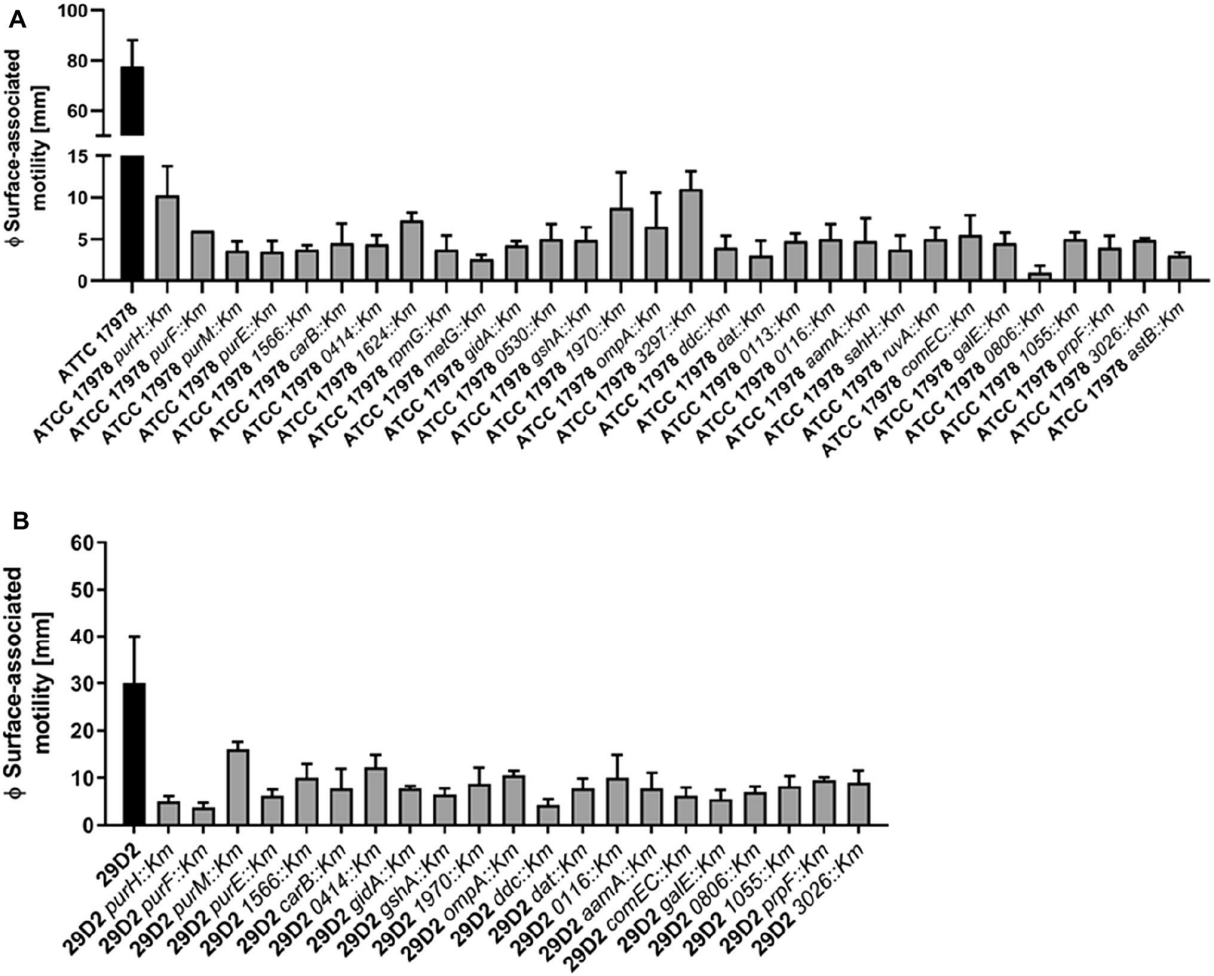
ATCC 17978 mutants (**a**) and 29D2 mutants (**b**) deficient in surface-associated motility. Wildtypes and mutants of strains ATCC 17978 and 29D2 were inoculated on motility plates. Plates were incubated for 16 h at 37 °C. The diameter (*Ø*) of surface-associated motility spreading zone was measured and triplicates were statistically analyzed. All mutants of strains ATCC 17978 (**a**) and 29D2 (**b**) display a significant motility deficiency compared to the respective parental strain

**Fig. 2:**
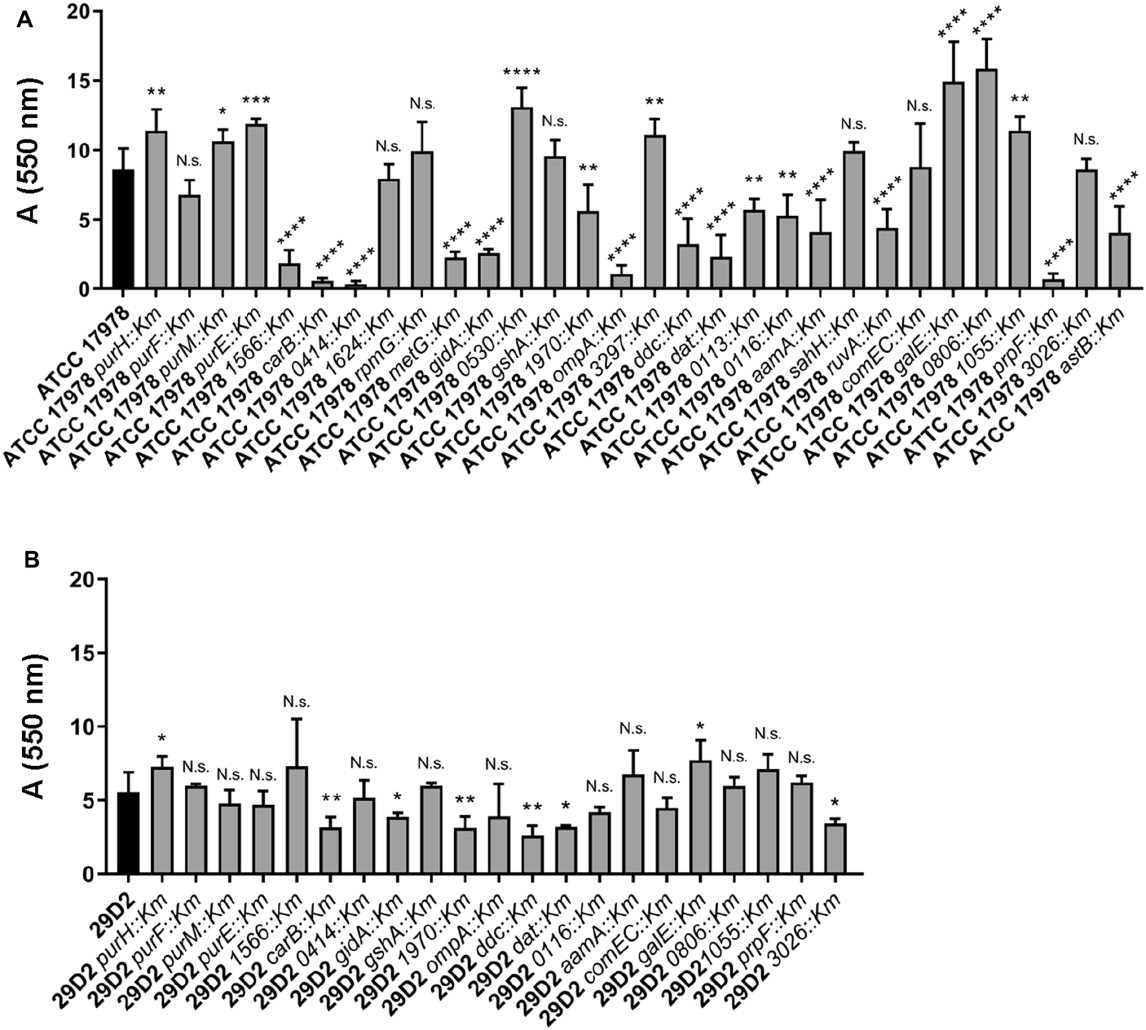
Pellicle biofilm formation of ATCC 17978 wildtype and mutants (**a**) and 29D2 wildtype and mutants (**b**). *A. baumannii* pellicle biofilms developed within 3 days of incubation at room temperature were stained with a 0.5% crystal violet solution for 20 min. The biofilm was scrubbed and flushed off the tube walls and absorption determined at 550 nm. For each strain three independent experiments were performed and statistical significance was analyzed by the Student’s *t* test (two-tailed, unpaired). Significance as indicated: *P value ≤ 0.05; **P value ≤ 0.01; ***P value ≤ 0.001; ****, *P* value ≤ 0.0001

To this end, surface-associated motility was analyzed on 0.5% agarose plates (Fig. 1a, Supplementary Table S3). All selected motility-deficient mutants of ATCC 17978 exhibited at least a sevenfold reduction of the spreading zone. Subsequently, DNA isolated from these transposon mutants was used to generate mutants in 29D2. All 29D2 mutants displayed a motility-deficient phenotype compared to the wildtype strain (Fig. 1b). Most ATCC 17978 mutants showed a 16-fold reduced surface-associated motility compared to the wildtype strain (Fig. 2a), whereas the *0806::Km* mutant lacked almost any measurable surface-associated motility. Three mutants, *purH::Km*, *1970::Km*, and *3297::Km*, showed tenfold reduced surface-associated motility. Most 29D2 mutants displayed a fourfold reduction in their surface-associated motility. The most pronounced reduction in motility appeared in mutants *purH::Km*, *purF::Km*, and *ddc::Km*. The mutant *purM::Km* had the lowest reduction in surface-associated motility.

To summarize, all mutations initially identified in ATCC 17978 that conferred motility defects were also found to cause motility-deficient phenotypes when introduced into the orthologous genes of 29D2.

### Pellicle Biofilm Formation

The formation of pellicle biofilms occurs at the air–liquid interface and is distinct from submerged biofilms [42, 47, 48]. A correlation between surface-associated motility and pellicle biofilm formation has been described for *A. baumannii* [42]. We examined the ability of our motility-deficient mutants to form pellicles within three days (Fig. 2, Table 1, Supplementary Table S4). A broad spread between low and high pellicle-producing mutants was visible, ranging between a 1.8-fold increase to more than a 25-fold decrease. For 15 of 30 mutants less than 67% of the wildtype-specific pellicle biomass was quantified (Table 1, Fig. 2a). In the mutants *carB::Km*, *0414::Km,* and *prpF::Km* a pellicle biomass less than 8% compared to the wildtype biomass was measured. This significant decrease was not observed by inactivation of the orthologous gene in the 29D2 background. In ATCC 17978, eight mutants were able to produce more pellicle biomass compared to the wildtype strain (Fig. 2a). 29D2 mutants only displayed small changes in pellicle biofilm formation compared to wildtype, with a range of the mutants’ pellicle biomass production from a 1.3-fold increase to a 2.1-fold decrease. Thirteen of 21 tested 29D2 mutants did not display any significant change in their pellicle biofilm formation compared to the parental strain (Table 2).

**Table 1:** Summary of experimental results on genes identified to be important for surface-associated motility in *A. baumannii* ATCC 17978

Locus tag in ATCC 17978	Annotation/gene name	Predicted function	Motility deficiency^a^	Growth deficiency^b^	Attenuation in *Galleria mellonella* infection^c^	Pellicle biofilm formation^d^	MIC values^e^		
							Ampicillin	Imipenem	Tetracycline
Purine/pyrimidine/folate biosynthesis									
A1S_2187	*purH*	Phosphoribosylaminoimidazolecarboxamide formyltransferase (purine synthesis)	+	Y	N.s.	↑**	N.s.	N.s.	N.s.
A1S_2251	*purF*	Amidophosphoribosyltransferase (purine synthesis)	++	Y	N.s.	N.s.	R***	N.s.	R*
A1S_2605	*purM*	Phosphoribosylaminoimidazole synthetase (purine synthesis)	+++	Y	*	↑*	R***	N.s.	S*
A1S_2964	*purE*	Phosphoribosylaminoimidazole carboxylase mutase subunit (purine synthesis)	+++	Y	N.s.	↑***	R***	S**	S*
A1S_1566		6-Pyruvoyl-tetrahydropterin synthase (folate biosynthesis)	+++	Y	**	↓****	R****	N.s.	N.s.
A1S_2687	*carB*	Carbamoylphosphate synthase subunit (pyrimidine synthesis)	++	Y	****	↓****	N.s.	S***	S**
Alarmones/ stress metabolism									
A1S_0414		Ap5A pyrophosphatase	++	Y	*	↓****	R****	N.s.	N.s.
A1S_1624		Ap4A hydrolase	++	Y	N.s.	N.s.	N.s.	S***	N.s.
RNA modification/ regulation									
A1S_0447	*rpmG*	50S ribosomal protein L33	+++	Y	N.s.	N.s.	N.s.	S*	S**
A1S_0778	*metG*	Methionyl-tRNA synthetase	+++	Y	****	↓****	N.s.	N.s.	S***
A1S_2182	*gidA*	Glucose-inhibited division protein A, FAD-binding protein	++	Y	N.s.	↓****	R*	N.s.	N.s.
Oxidative stress									
A1S_0530		Rhodanese domain-containing protein, sulfurtransferase	++	N	N.s.	↑****	N.s.	N.s.	N.s.
A1S_3366	*gshA*	Gamma-glutamate-cysteine ligase	++	Y	*	N.s.	R***	N.s.	N.s.
Outer membrane proteins									
A1S_1970		Outer membrane protein (Omp85 family)	++	Y	**	↓**	R****	S*	N.s.
A1S_2840	*ompA*	Outer membrane protein	++	N	****	↓****	N.s.	R***	S*
A1S_3297		Putative outer membrane protein	+	Y	**	↑**	R***	R*	N.s.
1,3-diaminopropane biosynthesis									
A1S_2453	*ddc*	l-2,4-Diaminobutyrate decarboxylase, biosynthesis of 1,3-diaminopropane (DAP)	+++	Y	N.s.	↓****	N.s.	N.s.	S***
A1S_2454	*dat*	l-2,4-Diaminobutyrate:2-ketoglutarate 4-aminotransferase, biosynthesis of 1,3-diaminopropane (DAP)	+++	Y	*	↓****	N.s.	N.s.	S***
Lipopeptide synthesis/ export									
A1S_0113		Acyl-CoA dehydrogenase (putative lipoprotein biosynthesis)	++	N	N.s.	↓**	R*	N.s.	N.s.
A1S_0116		RND superfamily transporter (efflux pump)	++	N	N.s	↓**	R***	N.s.	N.s.
DNA modification/repair/uptake									
A1S_0222	*aamA*	Adenine-specific methyltransferase	++	Y	***	↓****	N.s.	S***	S**
A1S_2334	*sahH*	*S*-Adenosyl-l-homocysteine hydrolase	+++	Y	*	N.s.	R***	N.s.	N.s.
A1S_2587	*ruvA*	Holliday junction helicase subunit A	++	Y	N.s.	↓****	R***	N.s.	S**
A1S_2610	*comEC*	Competence factor	++	N	**	N.s.	N.s.	N.s.	N.s.
Other									
A1S_0065	*galE*	UDP-glucose 4-epimerase, lipopolysaccharide biosynthesis	++	N	****	↑****	R***	N.s.	R*
A1S_0806		Adenosylmethionine-8-amino-7-oxononanoate aminotransferase	+++	Y	N.s.	↑****	R***	R****	N.s.
A1S_1055		Soluble lytic murein transglycosylase	++	N	**	↑**	N.s.	N.s.	S*
A1S_2761	*prpF*	2-Methylaconitate isomerase	+++	Y	N.s.	↓****	R*	N.s.	S**
A1S_3026		Hyp. secreted ribonuclease T2 (predicted secretion signal)	++	N	N.s	N.s.	R****	R***	N.s.
A1S_3129	*astB*	Succinylarginine dihydrolase	+++	Y	N.s.	↓****	R***	N.s.	S**

^a^Compared to ATCC 17978 wild type strain (WT); diameter of the spreading zone was measured: ‘+++, 0–4 mm; ‘++ < 4–9 mm; ‘+, <9 mm

^b^Comparison of bacterial growth curves. *Y* yes growth deficiency compared to WT was observed, *N* no growth deficiency was observed

^c^Compared to ATCC 17978 WT; unpaired *t* test was performed after 5 days p.i.: *N.s.* not significant, *P value ≤ 0.05; **P value ≤ 0.01; ***P value ≤ 0.001; ****P value ≤ 0.0001

^d^Compared to ATCC 17978 WT; unpaired *t* test was performed: *N.s.* not significant; *P value ≤ 0.05; **P value ≤ 0.01; ***P value ≤ 0.001; ****P value ≤ 0.0001; ↑ more than ATCC 17978 WT; ↓ less than ATCC 17978 WT

^e^Compared to ATCC 17978 WT; unpaired *t* test was performed: *N.s.* not significant; *P value ≤ 0.05; **P value ≤ 0.01; ***P value ≤ 0.001; ****P value ≤ 0.0001; *R* resistant, *S* susceptible

**Table 2:** Summary of experimental results on genes identified to be important for surface-associated motility in *A. baumannii* 29D2

Locus tag in ATCC 17978	Annotation/gene name	Motility deficiency^a^	Growth deficiency^b^	Attenuation in *Galleria mellonella* infection^c^	Pellicle biofilm formation^d^	MIC values^e^			
						Ampicillin	Imipenem		Tetracycline
Purine/pyrimidine/folate biosynthesis									
A1S_2187	*purH*	**++**	**Y**	*	↑*	S*		**N.s.**	**N.s.**
A1S_2251	*purF*	**++**	**Y**	**N.s.**	**N.s.**	S**		S*	N.s.
A1S_2605	*purM*	**+**	**Y**	N.s.	N.s.	S*		**N.s.**	N.s.
A1S_2964	*purE*	**+**	**Y**	**N.s.**	N.s.	S**		N.s.	N.s.
A1S_1566		**+**	**Y**	***	N.s.	S*		**N.s.**	S*
A1S_2687	*carB*	**+**	**Y**	****	↓**	S**		S**	N.s.
Alarmones/stress metabolism									
A1S_0414		**+**	N	N.s.	N.s.	N.s.		**N.s.**	**N.s.**
RNA modification/regulation									
A1S_2182	*gidA*	**+**	N	**N.s.**	↓*	S*		**N.s.**	**N.s.**
Oxidative stress									
A1S_3366	*gshA*	**+**	**Y**	N.s.	**N.s.**	S*		**N.s.**	**N.s.**
Outer membrane proteins									
A1S_1970		**+**	N	N.s.	↓**	N.s.	N.s.		**N.s.**
A1S_2840	*ompA*	**+**	Y	****	N.s.	S*	S**		N.s.
1,3-Diaminopropane biosynthesis									
A1S_2453	*ddc*	**++**	**Y**	*	↓**	S**	S*		N.s.
A1S_2454	*dat*	**+**	**Y**	N.s.	↓*	S**	**N.s.**		R*
Lipopeptide synthesis/export									
A1S_0116		**+**	**N**	**N.s.**	N.s.	N.s.	**N.s.**		**N.s.**
DNA modification/repair/uptake									
A1S_0222	*aamA*	**+**	N	*	N.s.	**N.s.**	R**		N.s.
A1S_2610	*comEC*	**+**	Y	****	**N.s.**	S**	S**		S*
Other									
A1S_0065	*galE*	**++**	Y	****	↑*	S*	**N.s.**		N.s.
A1S_0806		**+**	N	**N.s.**	N.s.	S*	S****		**N.s.**
A1S_1055		**+**	**N**	**	N.s.	**N.s.**	S*		N.s.
A1S_2761	*prpF*	**+**	**Y**	**	N.s.	S*	S*		S*
A1S_3026		**+**	**N**	**	↓*	R*	R***		**N.s.**

A bold indicates concordance to results obtained for strain ATCC 17978

^a^Compared to 29D2 wild type strain (WT); diameter of the spreading zone was measured: +++, 0–3 mm; ++, < 3–6 mm; +, <6 mm

^b^Comparison of bacterial growth curves. *Y* growth deficiency compared to WT was observed, *N* no growth deficiency was observed

^c^Compared to 29D2 WT; unpaired *t* test was performed after 5 days p.i.: *n.s.* not significant; * P value ≤ 0.05; **P value ≤ 0.01; ***P value ≤ 0.001; ****P value ≤ 0.0001

^d^Compared to 29D2 WT; unpaired *t* test was performed: *n.s.* not significant; *P value ≤ 0.05; **P value ≤ 0.01; ***P value ≤ 0.001; ****P value ≤ 0.0001; ↑ more than 29D2 WT; ↓ less than 29D2 WT

^e^Compared to 29D2 WT; unpaired *t* test was performed: *n.s.* not significant; *P value ≤ 0.05; **P value ≤ 0.01; ***P value ≤ 0.001; ****P value ≤ 0.0001; *R* resistant, *S* susceptible

In summary, the ATCC 17978 parental strain produced more pellicle biofilms compared to 29D2. Conspicuous changes in biofilm formation could mainly be observed among ATCC 17978 mutants. Concordance of pellicle formation phenotypes between the mutants of both strains was limited suggesting that strain-specific traits that are independent of surface-associated motility influence pellicle biomass production.

### Bacterial Growth

**Fig. 3:**
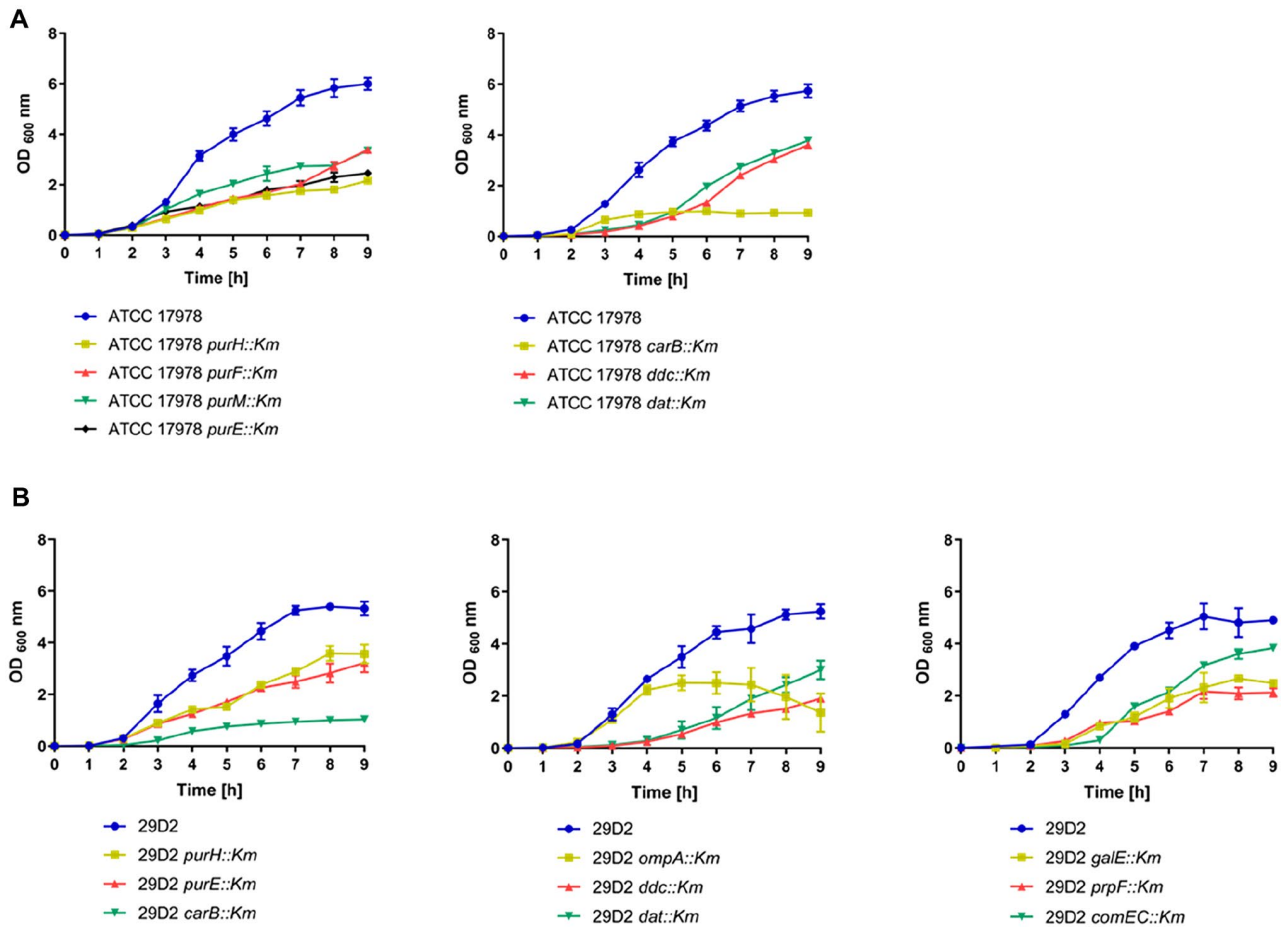
Growth deficiency of mutant strains from ATCC 17978 (**a**) and 29D2 (**b**) mutant libraries. OD-adjusted bacterial cultures were grown for 9 h at 37 °C under constant shaking. Every hour cultures were measured at an OD of 600 nm. For each strain data obtained from three independent cultures grown on the same day were averaged and represented by the mean ± SD. Growth defects compared to wildtype ATCC 17978 were observed for mutants that are involved in purine, pyrimidine, and diaminopropane biosynthesis (**a**). In strain 29D2, growth defects were observed for mutants involved in purine/pyrimidine/folate and diaminopropane biosynthesis, and for mutants *galE::Km*, *ompA::Km,* and *prpF::Km* (**b**). See Supplementary Figs. S4 and S5 for growth curves of all strains described in this study. Supplementary Table S5 provides data and analyses on endpoint measurements and Table 1 summarizes statistically significant differences in growth of mutants compared to wildtype strains based on endpoint measurements

The ability of motility-deficient mutants to grow as a planktonic culture under aeration was assayed. Growth curves and data for all tested strains are provided in Supplementary Fig. S4 (ATCC 17978 mutants), Supplementary Fig. S5 (29D2 mutants), and Supplementary Table S5. For ATCC 17978, 22 of 30 tested mutant strains exhibited significant growth defects compared to the parental strain (Table 1). The most striking growth defects (Fig. 3a) were observed in the mutants defective in purine biosynthesis (*purH::Km*, *purF::Km*, *purM::Km*, and *purE::Km*), pyrimidine biosynthesis (*carB::Km*), and diaminopropane biosynthesis (*ddc::Km* and *dat::Km*). Only 8 of 30 tested mutant strains were able to grow without any defect compared to the parental strain (Table 1). By testing the 29D2 mutant strains we observed 13 of 21 strains with notable planktonic growth defects (Table 2). Within this group most striking defects were observed with mutations associated with purine biosynthesis (*purH::Km*, *purF::Km*, *purM::Km*, and *purE::Km*), pyrimidine biosynthesis (*carB::Km*), folate biosynthesis (*1566::Km*), and diaminopropane biosynthesis (*ddc::Km* and *dat::Km*). Additionally, *galE::Km*, *comEC::Km,* and *prpF::Km* mutants displayed strong growth deficiencies (Fig. 3b). The mutant *ompA::Km* showed growth comparable to the parental strain for up to 4 h, reached a growth maximum of 2.5 ± 0.28 OD_600_ nm after 5 h, but then slowly collapsed to 1.36 ± 0.73 after 9 h. No growth defects were observed in 8 of 21 tested mutants (Table 2).

In summary, we found that genes involved in purine/pyrimidine and diaminopropane biosynthesis, oxidative stress, and propionate catabolism were crucial for growth of ATCC 17978 and 29D2 in LB medium.

### *Galleria mellonella* caterpillar infection

**Fig. 4:**
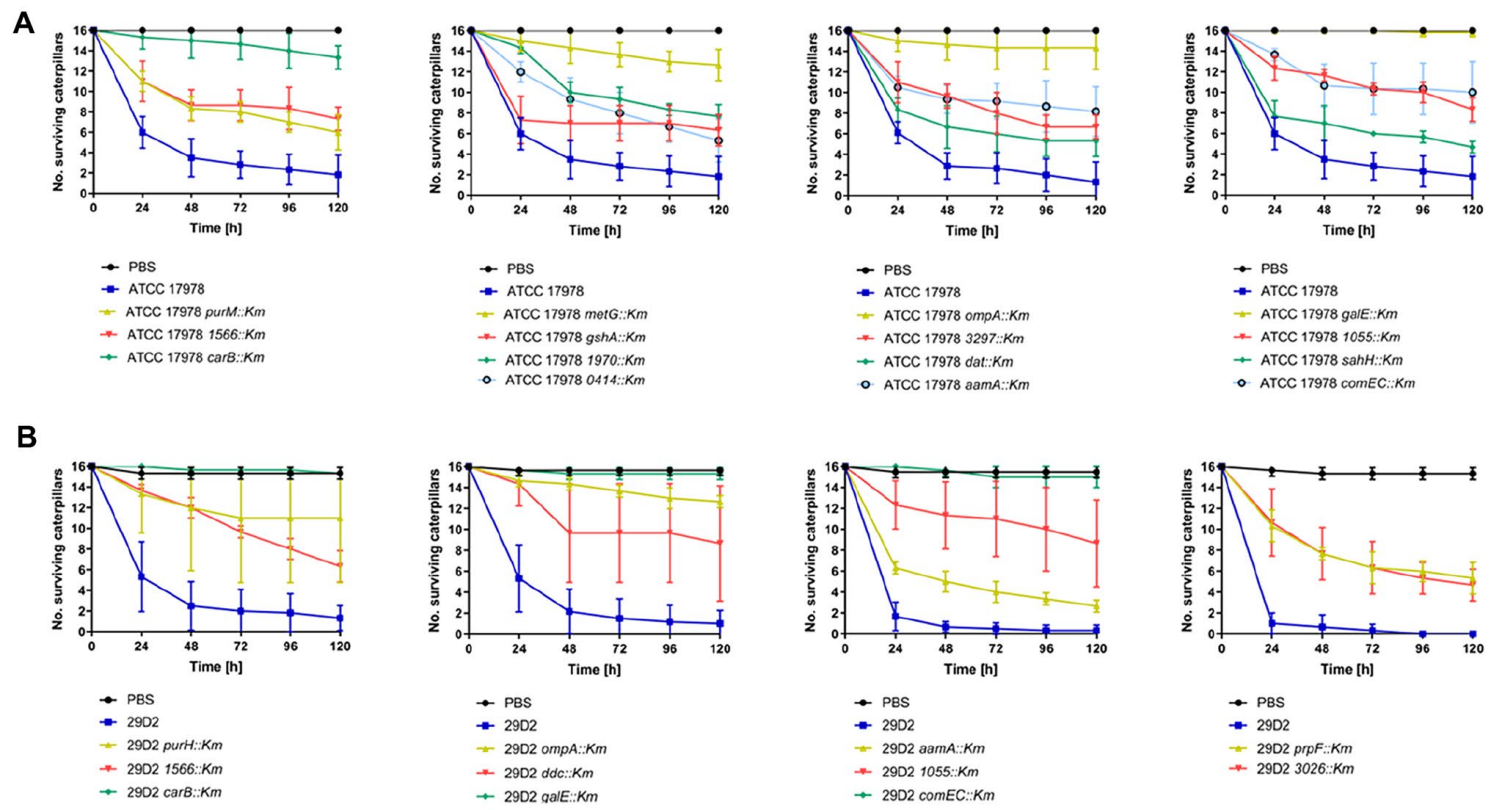
Attenuation of *A. baumannii* ATCC 17,978 mutants (**a**) and 29D2 mutants (**b**) in the *Galleria mellonella* caterpillar infection model. *G. mellonella* caterpillars were infected with 3 × 10^5^ CFU of *A. baumannii* strains as indicated. Sterile PBS (black lines) was used as a control. Three independent experiments were performed with groups of 16 caterpillars for every bacterial strain and control. Data obtained from three independent experiments were averaged and represented by the mean ± SD. In strain ATCC 17978, 15 out of 30 mutants show a significant attenuation 5 days p.i. (*P* values see Table 1) compared to the wildtype strain (**a**). In strain 29D2 11 out of 21 mutants are attenuated (*P* values indicated in Table 2) in the *Galleria mellonella* infection model (**b**). See Supplementary Figs. S6 and S7 for infection data of all strains described in this study

To gain insight into a possible correlation between motility and virulence we made use of the *G. mellonella* infection model. *G. mellonella* infection of ATCC 17978 wildtype and mutant strains as shown in Supplementary Fig. S6. Fifteen of 30 tested mutant strains displayed a significant attenuation in *G. mellonella* infection (Table 1). Another four mutant strains showed some attenuation but this was not significant. The remaining 11 mutant strains did not display attenuation (Supplementary Fig. S6, Table 1, Supplementary Table S6). Most pronounced attenuation was observed in strains *carB::Km*, *metG::Km*, *ompA::Km,* and *galE::Km* (Fig. 4a). These results suggest an important role for these genes in virulence. However, to exclude the possibility that attenuation could be due to decreased planktonic growth, we compared the caterpillar infection results to our bacterial growth data (Fig. 3, Supplementary Figs. S4, S5). Among the above mentioned mutants, only the *galE*::*Km* mutant was not significantly affected in growth. Overall, we found that for 11 of 15 significantly attenuated mutant strains the caterpillar infection data could possibly be influenced by decreased growth rates (Table 1, Fig. 3).

The *G. mellonella* infection with 29D2 wildtype and mutant strains data is shown in Supplementary Fig. S7. Eleven of 21 29D2 mutants were significantly attenuated in the *G. mellonella* infection model (Supplementary Fig S7, Table 2, Supplementary Table S7). Within this group the most pronounced attenuation was observed in strains *carB::Km*, *ompA::Km*, *galE::Km,* and *comEC::Km* (Fig. 4b). Eight of 11 significantly attenuated mutant strains manifested a growth deficiency compared to the parental strain (Supplementary Fig. S5, Table 2).

In summary, concordant infection traits were observed for 12 mutants of both strains including mutants affected in purine/pyrimidine/folate biosynthesis. Among these 12 strains, most significant attenuation was observed for *carB::Km*, *ompA::Km,* and *galE::Km*.

As a control, the CFUs were determined from the OD-adjusted bacterial cultures used for the infection experiments. Interestingly, for both ATCC 17978 *ompA::Km* and 29D2 *ompA::Km* mutants we observed 1–2 log scale lower CFU numbers compared to the OD-adjusted suspension. Subsequent microscopic examination revealed a distinct cell elongation or chain formation of both *ompA::Km* mutant strains compared to their parental strains (Supplementary Fig. S8).

### MIC Determination

We aimed to elucidate the correlation between motility-deficient mutants and their sensitivity to the bactericidal antibiotics ampicillin and imipenem as well as to the bacteriostatic antibiotic tetracycline. For ATCC 17978, 18 of 30 mutants displayed significant resistance to ampicillin compared to the parental strain (Tables 1, 3). The highest MIC values were obtained in mutant strains *0414::Km, 3026::Km*, and *1566::Km*. The only mutant strain which showed decreased resistance (0.7-fold decrease) to ampicillin was *aamA::Km*. By contrast, a significantly increased sensitivity to imipenem was observed in six of the tested strains. Furthermore, a significantly increased resistance to imipenem was observed in four of the tested mutants (*ompA::Km*, *3297::Km*, *0806::Km*, and *3026::Km*). For tetracycline, we found 13 of 30 mutants to be significantly more sensitive compared to the parental strain. Only 2 of 30 mutant strains, *purF::Km* and *galE::Km*, displayed significantly increased resistance to tetracycline. Analogous studies on 29D2 mutant strains revealed a significantly increased sensitivity to ampicillin in 15 of 21 tested mutant strains (Tables 2, 3). Only strain *3026::Km* was significantly increased in resistance to ampicillin, with a 1.7–fold increased MIC value. Another mutant strain with a 1.5-fold increased ampicillin MIC value, although not significant, was *aamA::Km*. Similar effects were observed for imipenem. Here, strains *3026::Km* and *aamA::Km* displayed significant resistance compared to the parental strain. Increased sensitivity to imipenem was observed in 8 of 21 tested mutants. For the MIC values of tetracycline, we found the 3 mutant strains *1566::Km*, *comEC::Km*, and *prpF::Km* to be significantly more susceptible. Only one mutant, *dat::Km*, was significantly more resistant to tetracycline with a 1.8-fold increase.

**Table 3:** Minimal inhibitory concentration (MIC) of ampicillin, tetracycline and imipenem determined from ATCC 17978 wildtype/mutants and 29D2 wildtype/mutants

Locus tag	Gene name	Ampicillin^a^		Imipenem^a^		Tetracycline^a^	
		ATCC 17978	29D2	ATCC 17978	29D2	ATCC 17978	29D2
Wildtype		25.3	36.5	0.23	0.25	2.1	3.25
A1S_2187	*purH*	32	***18.6***	0.23	0.21	1.6	1.5
A1S_2251	*purF*	**48**	***16.0***	0.25	***0.16***	**3.0**	2.3
A1S_2605	*purM*	**53.3**	***17.3***	0.21	0.18	***1.0***	2.3
A1S_2964	*purE*	**53.3**	***10.6***	***0.13***	0.21	***1.5***	2.0
A1S_1566		**96**	***18.6***	0.23	0.23	1.8	***0.9***
A1S_2687	*carB*	32	***3.0***	***0.10***	***0.03***	***0.9***	2.0
A1S_0414		**106.6**	32.0	0.23	0.25	1.6	2.0
A1S_1624		32	–	***0.125***	–	2.1	–
A1S_0447	*rpmG*	29.3	–	***0.16***	–	***1.0***	–
A1S_0778	*metG*	32	–	0.23	–	***0.46***	–
A1S_0530		24	–	0.19	–	2.0	–
A1S_3366	*gshA*	**85.3**	***26.6***	0.29	0.23	1.6	2.0
A1S_1970		**64**	32.0	***0.16***	0.25	2.5	1.8
A1S_2840	*ompA*	29.3	***13.3***	**0.46**	***0.13***	***1.5***	3.0
A1S_3297		**58.6**	–	**0.33**	–	2.0	–
A1S_2453	*ddc*	32	***5.0***	0.46	***0.14***	***0.38***	4.6
A1S_2454	*dat*	26.6	***5.3***	0.25	0.23	***0.29***	**6.0**
A1S_0113		**42.6**	–	0.25	–	2.0	–
A1S_0116		**85.3**	29.3	0.21	0.33	2.6	1.5
A1S_0222	*aamA*	18.6	42.6	***0.125***	**0.38**	***0.9***	2.6
A1S_0065	*galE*	**53.3**	***13.3***	0.23	0.18	**3.0**	2.0
A1S_0806		**53.3**	***13.3***	**0.38**	***0.023***	2.0	1.6
A1S_1055		26.6	21.3	0.29	***0.14***	***1.3***	1.6
A1S_2182	*gidA*	**37.3**	***24.0***	0.19	0.25	3.0	4.0
A1S_2334	*sahH*	**74.6**	–	0.18	–	1.8	–
A1S_2587	*ruvA*	**53.3**	–	0.19	–	***1.0***	–
A1S_2610	*comEC*	29.3	***5.3***	0.23	***0.10***	1.6	***0.6***
A1S_2761	*prpF*	**42.6**	***14.6***	0.25	***0.16***	***0.8***	***0.5***
A1S_3026		**106.6**	**64.0**	**0.42**	**0.46**	2.6	1.5
A1S_3129	*astB*	**74.6**	–	0.23	–	***1.1***	–

A bold indicates that MIC values of mutant strains are significantly resistant compared to the wildtype and a bold italic indicates susceptibility

^a^Averaged MIC values in (µg/mL) determined from three independent experiments. ‘–’ indicates ‘not tested’

In conclusion, mutants from the 29D2 background predominantly showed increased sensitivity to all tested antibiotics. By contrast, many mutants of ATCC 17978 showed increased resistance to ampicillin, but increased sensitivity to imipenem and tetracycline.

## Discussion

**Fig. 5:**
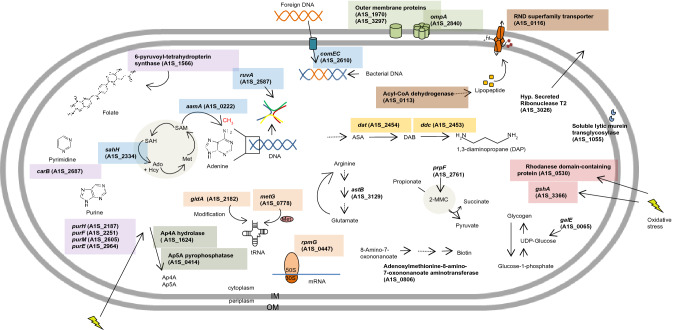
Genes inactivated in *A. baumannii* ATCC 17978 mutants with a surface-associated motility defect and their predicted/putative function in the bacterial cell. The color code indicates that these mutants belong to the same functional, processual and/or structural category. *OM* outer membrane, *IM* inner membrane, *Ap4A* diadenosine tetraphosphate, *Ap5A* diadenosine pentaphosphate, *SAM* *S*-adenosyl-l-methionine, *SAH* *S*-adenosylhomocysteine, *Ado* adenosine, *Hcy* homocysteine, *Met* methionine, *ASA* l-aspartate 4-semialdehyde, *DAB* l-2,4-diaminobutanoate, *2-MMC* 2-methylcitric acid cycle

Here, we identified and characterized 30 genes involved in *A. baumannii* surface-associated motility with respect to bacterial growth, pellicle biofilm formation, virulence, and antibiotic resistance. We discuss motility-deficient mutants with regards to their known/putative gene function in the bacterial cell (Fig. 5; Supplementary Table S8).

### Purine/Pyrimidine/Folate Biosynthesis

We identified four proteins involved in purine (*pur*) biosynthesis to be essential for *A. baumannii* surface-associated motility: PurH, PurF, PurM, and PurE. In *A. nosocomialis* M2, inactivation of *purK* (*a1s_2963*) has been previously described to result in a 70% reduction in surface motility [26]. Mutations in the genes *purD*, *purF*, *purH*, *purL*, and *purM* abolished K^+^-dependent colony spreading in *Bacillus subtilis* [49]. The *pur* genes were also demonstrated to be essential for biofilm formation in *Bacillus cereus* [50–52]. Interestingly, our study revealed no defective role of *pur* genes in pellicle biofilm formation. In contrast, mutations *purH::Km*, *purM::Km*, and *purE::Km* in ATCC 17978 and *purH::Km* in 29D2 produced significantly more pellicle biomass than their parental strains. A pellicle proteome study in ATCC 17978 showed that the Pur proteins were differentially expressed under planktonic and pellicle growth conditions [47].

In addition, we found all tested *pur* mutants to display growth defects in LB media. For various bacteria, *pur* genes were identified to be required for growth in human serum [53, 54]. Due to the fact that all *pur* mutants displayed growth defects we expected these mutants to be attenuated in the *G. mellonella* infection, but we only found mutants ATCC 17978 *purM::Km* and 29D2 *purH::Km* had significant attenuation. Purine biosynthesis mutants in *Burkholderia cenocepacia* were also found to be attenuated in the *G. mellonella* infection model as well as in *C. elegans* and *D. melanogaster* infections [55]. De novo purine biosynthesis has also been shown to be required for virulence in ATCC 17978 in the mouse lung [56], and in several other bacteria [57–59].

The *A. baumannii* gene *a1s_2687* encodes the large subunit (*carB*) of carbamoylphosphate synthase which is required for the de novo synthesis of arginine and pyrimidines [60]. Pyrimidines are known to be involved in biofilm formation in *P. aeruginosa* [61] and *E. coli* [62]. In *A. baumannii*, inactivation of *carB* caused significantly decreased persistence in a mouse pneumonia model [56]. The contribution of *carB* to *A. baumannii* virulence was confirmed by our results. Inactivation of *carB* in ATCC 17978 and 29D2 resulted in the greatest phenotypic alterations in planktonic growth, pellicle biofilm formation, and *G. mellonella* caterpillar infection of all tested mutants. Interestingly, similar observations were also made for a knockout of *carB* in *Xanthomonas citri*, which was decreased in swimming motility, biofilm formation, and growth [63]. CarB was also found to be required for growth of *E. coli* in human serum [54]. A motility-deficient phenotype was identified for the gene *a1s_1566* (putative 6-pyruvoyl-tetrahydropterin synthase), involved in folate biosynthesis and thus crucial for biosynthesis of purines and deoxythymidine monophosphate. Further, we observed an involvement in virulence, bacterial growth, and pellicle biofilm formation. Taken together, these findings suggest that purine and pyrimidine genes contribute to important bacterial processes like motility, bacterial growth, pellicle formation, and virulence in *Acinetobacter* as has been shown before for other genera.

### Alarmone/Damage Metabolism

The *A. baumannii* genes *a1s_0414* and *a1s_1624* encode for an Ap5A pyrophosphatase and an Ap4A hydrolase (ApaH-like), respectively, and are proposed to be involved in depletion of putative alarmone/signaling molecules [64, 65] and/or damage metabolites [66, 67]. Recent work suggests that dinucleoside polyphosphates can be used by RNA polymerases to initiate transcription and to act as 5′-RNA caps that may stabilize RNA, while ApaA-like hydrolases are able to remove these caps [68]. The Ap4A hydrolase mutant seems to play a role in *A. baumannii* surface motility and planktonic growth. An *E. coli* Ap4A hydrolase (*apaH*) knockout mutant was previously associated with decreased motility [69]. In *Salmonella enterica* adhesion and invasion capacity into epithelial cells was reduced for the Δ*apaH* mutant [70]. In general, Ap4A and Ap5A are thought to be synthesized by aminoacyl-tRNA synthetases in the absence of tRNAs [71, 72]. Providing a possible link, we found a methionyl-tRNA synthetase in our surface motility-deficient library (discussed below).

### RNA Modification/Regulation

We found three genes involved in regulation and/or modification of RNAs: *metG* (methionyl-tRNA synthetase, *a1s_0778*), *rpmG* (50S ribosomal protein L33, *a1s_0447*), and *gidA* (glucose-inhibited division protein A, a tRNA modification enzyme, *a1s_2182*). The deficiency in motility of *gidA* mutants has been described for swarming motility in bacteria like *Bacillus cereus* [51], *Serratia* species SCBI [73], and *Pseudomonas syringae* [74]. In the present study, a *gidA* null allele in strains ATCC 17978 and 29D2 resulted in small decreases in their planktonic growth. Contrary results for Δ*gidA* bacterial growth have been reported [75]. Interestingly, proteomic analysis of *A. baumannii* planktonic and biofilm growth identified GidA only under biofilm growth conditions [76], while several studies reported the negative effect of *gidA* mutants on biofilm formation in different bacteria [77, 78]. In the present study we also saw a significant reduction in pellicle formation in both *gidA::Km* mutants (Fig. 2). An essential role of *gidA* in pellicle formation was also shown in *Bacillus cereus* [51]. While several GidA-associated virulence effects have been reported [75] we did not see significant attenuation in the *G. mellonella* infection model.

In contrast, the knockout of *metG* was associated with a significant attenuation in the *G. mellonella* infection model. Similarly, involvement of *metG* in *A. baumannii* virulence was also shown in a mouse pneumonia model [56]. The *metG::Km* mutant revealed a significantly reduced ability to form pellicles. In line, MetG was found to be more abundant in *A. baumannii* pellicle cells than in planktonic cells [47]. Here, we found the *metG::Km* mutant to be more sensitive to tetracycline, which agrees with observations of amino acid substitutions of MetG associated with increased antibiotic tolerance in *Burkholderia thailandensis* [79] and *E. coli* [80, 81].

We observed increased sensitivity of the *rpmG::Km* mutant to imipenem and tetracycline. These data are in line with a study which showed that a mitomycin C resistance phenotype was associated with RpmG overproduction in *E. coli* [82].

### Oxidative Stress Response

The ATCC 17978 gene *a1S_3366* is predicted to encode a gamma-glutamate-cysteine ligase (*gshA*) required to synthesize the antioxidant glutathione [83, 84]. Different studies observed decreasing swarming [85], swimming [85, 86], and twitching motility [86] of the *P. aeruginosa* Δ*gshA* mutant compared to the parental strain. Contrary results were found for the ability *of P. aeruginosa* Δ*gshA* to form biofilms (increased in [86] and decreased in [85]). We did not find any changes in pellicle biofilm production compared to the parental strains for both of our *gshA* mutants. In *Acinetobacter baylyi* the knockout of *gshA* increased sensitivity to metronidazole and ciprofloxacin [87]. We observed an enhanced sensitivity to ampicillin for the 29D2 *gshA::Km* mutant, but the ATCC 17978 *gshA::Km* mutant showed a resistant phenotype. Attenuation in *G. mellonella* infection was observed for the ATCC 17978 *gshA* mutant strain, which agrees with other studies describing *gshA* mutants to be attenuated in *C. elegans* infection (*P. aeruginosa* [88]) and a *Salmonella* murine infection model [89]).

The *A. baumannii* gene *a1s_0530* encodes a putative sulfurtransferase, supposed to be involved in oxidative stress detoxification and sulfur metabolism [90–93]. The significant increase in pellicle production of the corresponding mutant in ATCC 17978 fits with the involvement of oxidative stress response proteins in pellicles observed in a proteomic study of ATCC 17978 [47].

### Outer Membrane Proteins

The gene *a1s_3297* encodes a putative outer membrane protein (OMP) and *a1s_1970* encodes a putative membrane-associated Zn-dependent protease (RseP). Here we show that both genes are involved in *A. baumannii* virulence, pellicle formation, and antimicrobial resistance.

We found OmpA to be involved in *A. baumannii* surface-associated motility, which has been described for *A. nosocomialis* M2 [26]. Several studies have reported the involvement of OmpA in biofilm formation [94–96] and OmpA, along with other OMPs, was observed to accumulate in *A. baumannii* pellicle cells compared to planktonic cells [47]. We found the *ompA* knockout associated with a significant decrease in pellicle formation in ATCC 17978 but not in 29D2. For *A. baumannii* and a number of other pathogens, OmpA has been identified as a virulence factor [97–99]. In our study, the knockout of *ompA* in both tested strains significantly decreased the mutant’s ability to kill *G. mellonella*. We found the *ompA::Km* mutants exhibiting filamentous cell phenotypes in contrast to the parental strains. Filamentous cell morphologies are known to provide bacterial survival advantages, e.g. protection against phagocytosis, resistance against antibiotics, and enhanced response to environmental cues like quorum sensing [100]. In other bacteria, the loss of OmpA-like proteins resulted in reduced membrane integrity and alterations in cell division [101, 102]. OmpA is involved in the ability of *A. baumannii* to grow and persist in human serum [11, 103] and in the adherence and invasion of epithelial cells [104].

### Biosynthesis of 1,3-Diaminopropane

As previously shown, mutations in the genes *dat* and *ddc* abolished surface-associated motility, but can be restored by supplementation with 1,3-diaminopropane [35]. In the present study, we observed motility deficiency for these genes in 29D2. We also gained new insight into the pleiotropic effects of the *dat::Km* and *ddc::Km* mutants, such as a significant decrease in pellicle formation. This observation might represent species-specific traits as we see this effect in both tested strains.

### Lipopeptide Synthesis/Export

The genes *a1s_0113* and *a1s_0116* are involved in synthesis and export of a lipopeptide and are part of an operon consisting of 8 genes [26, 34]. The knockout of *a1s_0113* (acyl-CoA dehydrogenase) in *A. nosocomialis* M2 resulted in a significant surface motility defect [26], which correlates with our observation in ATCC 17978. Additionally, other genes of this operon have been reported to be necessary for motility [42], pellicle biofilm formation [34, 42], and biofilm formation on abiotic surfaces [34, 105]. A pellicle proteome analysis in ATCC 17978 found the proteins A1S_0112-A1S_0118, except A1S_0114, to accumulate in the pellicle [47]. Since the gene *a1s_0116* encodes an RND superfamily transporter, it may thus play a role in multi-drug resistance. Deletion of *a1s_0116* in ATCC 17978 resulted in significantly increased ampicillin resistance compared to the parental strain whereas no differences were observed with imipenem and tetracycline. A transcriptomic study on imipenem-resistant ATCC 17978 showed decreased expression of genes from the *a1s_0112-a1s_0119* cluster [106]. Clemmer et al. speculated that the lipopeptide synthesized from the *a1s_0112-a1s_0119* operon may act as a surfactant to promote motility [26]. While we could not show a significant effect of *a1s_0113* or *a1s_0116* inactivation on virulence in *G. mellonella*, significant attenuation was observed in the same model for an *a1s_0114* mutant [34]. No essential role of any of the *a1s_0112*-*a1s_0119* genes in virulence was also found for strain AB5075 [107]. In conclusion, our data confirm findings by other groups [26, 34, 42, 47, 105] indicating that genes of the *a1s_0112*-*a1s_0119* operon are essential for surface motility and pellicle formation in *A. baumannii*.

### DNA Modification/Repair/Uptake

Our library includes four mutant genes involved in DNA modification, uptake, and recombination. The gene *a1s_0222*, designated as *aamA,* encodes an orphan Type II N6-adenine DNA methyltransferase [108, 109]. Methylation is important for the regulation of various physiological processes [110, 111]. The prototypic orphan DNA adenine methyltransferase Dam has been shown to affect motility, virulence, and other traits in several bacteria [112, 113].

The *A. baumannii* gene *a1s_2334* encodes an *S*-adenosyl-l-homocysteine hydrolase (*sahH*), which takes part in the recycling of *S*-adenosyl-l-methionine. We show that inactivation of *sahH* in *A. baumannii* leads to strong motility deficiency, significant attenuation in *G. mellonella* caterpillar infection and increased antibiotic resistance. Furthermore, we found the Holliday junction helicase subunit A (*ruvA*/ *a1s_2587*) to be important for *A. baumannii* surface-associated motility, pellicle biofilm formation, and antibiotic resistance in ATCC 17978.

We identified the gene *a1s_2610* in our screening. Designated as *comEC*, this gene is involved in DNA uptake and incorporation of exogenous DNA into the genome. Phenotypically, a linkage between motility and natural transformation competence was shown in that *A. baumannii* can take up DNA while moving along wet surfaces [16, 44]. Genetically this interrelationship was illustrated by abolished twitching motility and natural transformation competence of *comEC* knockout mutants in different *A. baumannii* strains, and a defect in surface-associated motility was ascribed for the ATCC 17978 *comEC::Km* mutant [16]. Our results confirmed surface-associated motility deficiency in the 29D2 *comEC::Km* mutant strain. Deficiency in twitching motility was also shown for Δ*comEC* in *Thermus thermophilus* [114]. Here, a striking attenuation in *G. mellonella* caterpillar infection for the *comEC::Km* mutants in both strains was observed, similar to attenuation of *comEC::Km* mutant derivatives of various *A. baumannii* strains [16]. In *Listeria monocytogenes*, *comEC* was demonstrated to be involved in phagosomal escape, intracellular growth, and virulence [115]. However, *com* genes have been reported to be involved in biofilm formation [116], which we could not confirm for our *comEC::Km* mutant strains.

### Other Genes

A summary on additional genes identified here and their linkages to the literature is provided in Supplementary Table S8.

### *Galleria mellonella* Caterpillar Infection

A previous study analyzed 250,000 *A. baumannii* AB5075 transposon mutants for growth within *G. mellonella* larvae, and identified 300 genes essential for growth [107]. When comparing with these results, we could not identify concordant genes in our library, but we found that main categories of genes do match. For example, we found *galE* to be essential and in AB5075 numerous genes involved in structure and function of the cell envelope were found to be required for growth in *G. mellonella* [107]. Conversely, for example, the *gidA∷Km* mutant was not attenuated in *G. mellonella* infection in our study, but was stated to be essential for growth of AB5075 in *G. mellonella* [107]. It is known that AB5075 is more virulent than ATCC 17978 [107, 117], therefore, comparative studies are needed to unravel strain-specific and species-specific traits.

### Limitations

While our study highlights the need for comparative studies of specific mutant phenotypes in different strains to distinguish strain-specific from species-specific traits, it is clear that the two strains studied in detail here do not provide a sufficient basis to deduce such insight. Such comparative studies in combination with genome-based analyses may pave the way for the identification of species-specific traits and, ultimately, novel target sites.

The use of marker-based mutagenesis and naturally competent strains to efficiently generate sets of mutants in different strains has its shortcomings as recombination events are not necessarily limited to the site of the marker gene. Apart from homology-based recombination events, transfer of mobile genetic elements and even illegitimate recombination events may occur [118, 119] and could corrupt the mutants’ phenotypes. A few of the mutations described in this study have been partially characterized previously using additional strains (e.g. *ddc*, *dat*, *comEC, aamA* [16, 35, 108]). However, repetitive construction of the same mutants did not lead to significant phenotype variation arguing against a high frequency of such corrupting side-effects.

Complementation experiments and site-specific deletion mutagenesis would help to exclude polar effects of the transposon insertions and to verify the contribution of each gene. In support of the specificity of our findings, we found many groups of related mutants (e.g. purine and pyrimidine biosynthesis) and identified multiple linkages to motility mutants described in other organisms.

We could not achieve a saturated mutant library which indicates that surface-associated motility is probably under control of additional genes yet to be discovered.

## Conclusion

In this study, we identified 30 genes involved in surface-associated motility. All tested mutants originally identified as motility-deficient in strain ATCC 17978 also displayed a motility-deficient phenotype in strain 29D2. Some of these genes have already been linked to motility in *A. baumannii* (e.g. *comEC*, *a1s_0113,* and *a1s_0116*) or other bacteria (e.g. *carB* and *gidA*), but some of our findings represent new insights into requirements for surface-associated motility. Furthermore, we analyzed these mutants with respect to bacterial growth, pellicle biofilm formation, virulence in *G. mellonella* infection, and antibiotic resistance and used the naturally competent strain 29D2 to indicate whether the mutations showed strain-specific or species-specific traits. In summary, we can state that mutations in genes involved in purine/pyrimidine/folate biosynthesis are essential for all tested categories. Mutants that targeted RNA modification/regulation seem to mainly play a role in motility and pellicle formation. The discovery of novel genes required for surface-associated motility in *A. baumannii* demonstrates that more work is required to further define its genetic basis.

## Supplementary Information

Below is the link to the electronic supplementary material.

Supplementary file 1 (DOCX 5213 KB)

## References

[1] Baumann P (1968). Isolation of Acinetobacter from soil and water. J Bacteriol.

[2] Peleg AY, Seifert H, Paterson DL (2008). *Acinetobacter baumannii*: emergence of a successful pathogen. Clin Microbiol Rev.

[3] Harding CM, Hennon SW, Feldman MF (2018). Uncovering the mechanisms of *Acinetobacter baumannii* virulence. Nat Rev Microbiol.

[4] Vincent JL, Rello J, Marshall J, Silva E, Anzueto A, Martin CD, Moreno R, Lipman J, Gomersall C, Sakr Y, Reinhart K (2009). International study of the prevalence and outcomes of infection in intensive care units. JAMA.

[5] Landman D, Bratu S, Kochar S, Panwar M, Trehan M, Doymaz M, Quale J (2007). Evolution of antimicrobial resistance among *Pseudomonas aeruginosa*, *Acinetobacter baumannii* and *Klebsiella pneumoniae* in Brooklyn. NY J Antimicrob Chemother.

[6] Tognim MC, Andrade SS, Silbert S, Gales AC, Jones RN, Sader HS (2004). Resistance trends of *Acinetobacter* spp. in Latin America and characterization of international dissemination of multi-drug resistant strains: five-year report of the SENTRY Antimicrobial Surveillance Program. Int J Infect Dis.

[7] Giammanco A, Cala C, Fasciana T, Dowzicky MJ (2017). Global assessment of the activity of tigecycline against multidrug-resistant gram-negative pathogens between 2004 and 2014 as part of the tigecycline evaluation and surveillance trial. mSphere.

[8] Tacconelli E, Carrara E, Savoldi A, Harbarth S, Mendelson M, Monnet DL, Pulcini C, Kahlmeter G, Kluytmans J, Carmeli Y, Ouellette M, Outterson K, Patel J, Cavaleri M, Cox EM, Houchens CR, Grayson ML, Hansen P, Singh N, Theuretzbacher U, Magrini N, Group WHOPPLW (2018). Discovery, research, and development of new antibiotics: the WHO priority list of antibiotic-resistant bacteria and tuberculosis. Lancet Infect Dis.

[9] Roca I, Espinal P, Vila-Farres X, Vila J (2012). The *Acinetobacter baumannii* oxymoron: commensal hospital dweller turned pan-drug-resistant menace. Front Microbiol.

[10] Giannouli M, Antunes LC, Marchetti V, Triassi M, Visca P, Zarrilli R (2013). Virulence-related traits of epidemic *Acinetobacter baumannii* strains belonging to the international clonal lineages I-III and to the emerging genotypes ST25 and ST78. BMC Infect Dis.

[11] Antunes LC, Imperi F, Carattoli A, Visca P (2011). Deciphering the multifactorial nature of *Acinetobacter baumannii* pathogenicity. PLoS ONE.

[12] Jawad A, Seifert H, Snelling AM, Heritage J, Hawkey PM (1998). Survival of *Acinetobacter baumannii* on dry surfaces: comparison of outbreak and sporadic isolates. J Clin Microbiol.

[13] Wagenvoort JH, Joosten EJ (2002). An outbreak *Acinetobacter baumannii* that mimics MRSA in its environmental longevity. J Hosp Infect.

[14] Greene C, Vadlamudi G, Newton D, Foxman B, Xi C (2016). The influence of biofilm formation and multidrug resistance on environmental survival of clinical and environmental isolates of *Acinetobacter baumannii*. Am J Infect Control.

[15] Eijkelkamp BA, Stroeher UH, Hassan KA, Elbourne LD, Paulsen IT, Brown MH (2013). H-NS plays a role in expression of *Acinetobacter baumannii* virulence features. Infect Immun.

[16] Wilharm G, Piesker J, Laue M, Skiebe E (2013). DNA uptake by the nosocomial pathogen *Acinetobacter baumannii* occurs during movement along wet surfaces. J Bacteriol.

[17] Harding CM, Tracy EN, Carruthers MD, Rather PN, Actis LA, Munson RS (2013). *Acinetobacter baumannii* strain M2 produces type IV pili which play a role in natural transformation and twitching motility but not surface-associated motility. MBio.

[18] Harshey RM (2003). Bacterial motility on a surface: many ways to a common goal. Annu Rev Microbiol.

[19] Jarrell KF, McBride MJ (2008). The surprisingly diverse ways that prokaryotes move. Nat Rev Microbiol.

[20] Mattick JS (2002). Type IV pili and twitching motility. Annu Rev Microbiol.

[21] Merz AJ, So M, Sheetz MP (2000). Pilus retraction powers bacterial twitching motility. Nature.

[22] Skerker JM, Berg HC (2001). Direct observation of extension and retraction of type IV pili. Proc Natl Acad Sci USA.

[23] Wall D, Kaiser D (1999). Type IV pili and cell motility. Mol Microbiol.

[24] Henrichsen J (1984). Not gliding but twitching motility of *Acinetobacter calcoaceticus*. J Clin Pathol.

[25] McBride MJ (2010). Shining a light on an opportunistic pathogen. J Bacteriol.

[26] Clemmer KM, Bonomo RA, Rather PN (2011). Genetic analysis of surface motility in *Acinetobacter baumannii*. Microbiology.

[27] Henrichsen J, Blom J (1975). Correlation between twitching motility and possession of polar fimbriae in *Acinetobacter calcoaceticus*. Acta Pathol Microbiol Scand B.

[28] Eijkelkamp BA, Stroeher UH, Hassan KA, Papadimitrious MS, Paulsen IT, Brown MH (2011). Adherence and motility characteristics of clinical *Acinetobacter baumannii* isolates. FEMS Microbiol Lett.

[29] Barker J, Maxted H (1975). Observations on the growth and movement of Acinetobacter on semi-solid media. J Med Microbiol.

[30] Mussi MA, Gaddy JA, Cabruja M, Arivett BA, Viale AM, Rasia R, Actis LA (2010). The opportunistic human pathogen *Acinetobacter baumannii* senses and responds to light. J Bacteriol.

[31] Eijkelkamp BA, Hassan KA, Paulsen IT, Brown MH (2011). Investigation of the human pathogen *Acinetobacter baumannii* under iron limiting conditions. BMC Genomics.

[32] McQueary CN, Kirkup BC, Si Y, Barlow M, Actis LA, Craft DW, Zurawski DV (2012). Extracellular stress and lipopolysaccharide modulate *Acinetobacter baumanni*i surface-associated motility. J Microbiol.

[33] Pérez-Varela M, Tierney ARP, Kim JS, Vázquez-Torres A, Rather P (2020). Characterization of RelA in *Acinetobacter baumannii*. J Bacteriol.

[34] Rumbo-Feal S, Perez A, Ramelot TA, Alvarez-Fraga L, Vallejo JA, Beceiro A, Ohneck EJ, Arivett BA, Merino M, Fiester SE, Kennedy MA, Actis LA, Bou G, Poza M (2017). Contribution of the *A. baumannii* A1S_0114 gene to the interaction with eukaryotic cells and virulence. Front Cell Infect Microbiol.

[35] Skiebe E, de Berardinis V, Morczinek P, Kerrinnes T, Faber F, Lepka D, Hammer B, Zimmermann O, Ziesing S, Wichelhaus TA, Hunfeld KP, Borgmann S, Grobner S, Higgins PG, Seifert H, Busse HJ, Witte W, Pfeifer Y, Wilharm G (2012). Surface-associated motility, a common trait of clinical isolates of *Acinetobacter baumannii*, depends on 1,3-diaminopropane. Int J Med Microbiol.

[36] Corral J, Pérez-Varela M, Barbé J, Aranda J (2020). Direct interaction between RecA and a CheW-like protein is required for surface-associated motility, chemotaxis and the full virulence of *Acinetobacter baumannii* strain ATCC 17978. Virulence.

[37] Jacobs AC, Blanchard CE, Catherman SC, Dunman PM, Murata Y (2014). An ribonuclease T2 family protein modulates *Acinetobacter baumannii* abiotic surface colonization. PLoS ONE.

[38] Heindorf M, Kadari M, Heider C, Skiebe E, Wilharm G (2014). Impact of *Acinetobacter baumannii* superoxide dismutase on motility, virulence, oxidative stress resistance and susceptibility to antibiotics. PLoS ONE.

[39] Ahmad I, Nygren E, Khalid F, Myint SL, Uhlin BE (2020). A Cyclic-di-GMP signalling network regulates biofilm formation and surface associated motility of *Acinetobacter baumannii *17978. Sci Rep.

[40] Tipton KA, Rather PN (2017). An ompR-envZ two-component system ortholog regulates phase variation, osmotic tolerance, motility, and virulence in *Acinetobacter baumannii* strain AB5075. J Bacteriol.

[41] Tipton KA, Dimitrova D, Rather PN (2015). Phase-variable control of multiple phenotypes in *Acinetobacter baumannii* strain AB5075. J Bacteriol.

[42] Giles SK, Stroeher UH, Eijkelkamp BA, Brown MH (2015). Identification of genes essential for pellicle formation in *Acinetobacter baumannii*. BMC Microbiol.

[43] Wilharm G, Skiebe E, Higgins PG, Poppel MT, Blaschke U, Leser S, Heider C, Heindorf M, Brauner P, Jackel U, Bohland K, Cuny C, Lopinska A, Kaminski P, Kasprzak M, Bochenski M, Ciebiera O, Tobolka M, Zolnierowicz KM, Siekiera J, Seifert H, Gagne S, Salcedo SP, Kaatz M, Layer F, Bender JK, Fuchs S, Semmler T, Pfeifer Y, Jerzak L (2017). Relatedness of wildlife and livestock avian isolates of the nosocomial pathogen *Acinetobacter baumannii* to lineages spread in hospitals worldwide. Environ Microbiol.

[44] Godeux AS, Lupo A, Haenni M, Guette-Marquet S, Wilharm G, Laaberki MH, Charpentier X (2018). Fluorescence-based detection of natural transformation in drug-resistant *Acinetobacter baumannii*. J Bacteriol.

[45] Ahmad I, Karah N, Nadeem A, Wai SN, Uhlin BE (2019). Analysis of colony phase variation switch in *Acinetobacter baumannii* clinical isolates. PLoS ONE.

[46] Choi KH, Kumar A, Schweizer HP (2006). A 10-min method for preparation of highly electrocompetent *Pseudomonas aeruginosa* cells: application for DNA fragment transfer between chromosomes and plasmid transformation. J Microbiol Methods.

[47] Kentache T, Ben Abdelkrim A, Jouenne T, De E, Hardouin J (2017). Global dynamic proteome study of a pellicle-forming *Acinetobacter baumannii* strain. Mol Cell Proteomics.

[48] Nait Chabane Y, Marti S, Rihouey C, Alexandre S, Hardouin J, Lesouhaitier O, Vila J, Kaplan JB, Jouenne T, De E (2014). Characterisation of pellicles formed by *Acinetobacter baumannii *at the air-liquid interface. PLoS ONE.

[49] Kinsinger RF, Kearns DB, Hale M, Fall R (2005). Genetic requirements for potassium ion-dependent colony spreading in *Bacillus subtilis*. J Bacteriol.

[50] Yan F, Yu Y, Gozzi K, Chen Y, Guo JH, Chai Y (2017). Genome-wide investigation of biofilm formation in *Bacillus cereus*. Appl Environ Microbiol.

[51] Okshevsky M, Louw MG, Lamela EO, Nilsson M, Tolker-Nielsen T, Meyer RL (2018). A transposon mutant library of *Bacillus cereus* ATCC 10987 reveals novel genes required for biofilm formation and implicates motility as an important factor for pellicle-biofilm formation. Microbiologyopen.

[52] Vilain S, Pretorius JM, Theron J, Brozel VS (2009). DNA as an adhesin: *Bacillus cereus* requires extracellular DNA to form biofilms. Appl Environ Microbiol.

[53] Zhang X, de Maat V, Guzman Prieto AM, Prajsnar TK, Bayjanov JR, de Been M, Rogers MRC, Bonten MJM, Mesnage S, Willems RJL, van Schaik W (2017). RNA-seq and Tn-seq reveal fitness determinants of vancomycin-resistant *Enterococcus faecium* during growth in human serum. BMC Genomics.

[54] Samant S, Lee H, Ghassemi M, Chen J, Cook JL, Mankin AS, Neyfakh AA (2008). Nucleotide biosynthesis is critical for growth of bacteria in human blood. PLoS Pathog.

[55] Schwager S, Agnoli K, Kothe M, Feldmann F, Givskov M, Carlier A, Eberl L (2013). Identification of *Burkholderia cenocepacia* strain H111 virulence factors using nonmammalian infection hosts. Infect Immun.

[56] Wang N, Ozer EA, Mandel MJ, Hauser AR (2014). Genome-wide identification of *Acinetobacter baumannii* genes necessary for persistence in the lung. MBio.

[57] Polissi A, Pontiggia A, Feger G, Altieri M, Mottl H, Ferrari L, Simon D (1998). Large-scale identification of virulence genes from *Streptococcus pneumoniae*. Infect Immun.

[58] Jenkins A, Cote C, Twenhafel N, Merkel T, Bozue J, Welkos S (2011). Role of purine biosynthesis in *Bacillus anthracis* pathogenesis and virulence. Infect Immun.

[59] Fuller TE, Kennedy MJ, Lowery DE (2000). Identification of *Pasteurella multocida* virulence genes in a septicemic mouse model using signature-tagged mutagenesis. Microb Pathog.

[60] Charlier D, Nguyen Le Minh P, Roovers M (2018). Regulation of carbamoylphosphate synthesis in *Escherichia coli*: an amazing metabolite at the crossroad of arginine and pyrimidine biosynthesis. Amino Acids.

[61] Ueda A, Attila C, Whiteley M, Wood TK (2009). Uracil influences quorum sensing and biofilm formation in *Pseudomonas aeruginosa* and fluorouracil is an antagonist. Microb Biotechnol.

[62] Garavaglia M, Rossi E, Landini P (2012). The pyrimidine nucleotide biosynthetic pathway modulates production of biofilm determinants in *Escherichia col*i. PLoS ONE.

[63] Zhuo T, Rou W, Song X, Guo J, Fan X, Kamau GG, Zou H (2015). Molecular study on the carAB operon reveals that carB gene is required for swimming and biofilm formation in *Xanthomonas citri* subsp. citri. BMC Microbiol.

[64] Kisselev LL, Justesen J, Wolfson AD, Frolova LY (1998). Diadenosine oligophosphates (Ap(n)A), a novel class of signalling molecules?. FEBS Lett.

[65] Bochner BR, Lee PC, Wilson SW, Cutler CW, Ames BN (1984). Appppa and related adenylylated nucleotides are synthesized as a consequence of oxidation stress. Cell.

[66] Linster CL, Van Schaftingen E, Hanson AD (2013). Metabolite damage and its repair or pre-emption. Nat Chem Biol.

[67] Despotovic D, Brandis A, Savidor A, Levin Y, Fumagalli L, Tawfik DS (2017). Diadenosine tetraphosphate (Ap4A): an *E. coli* alarmone or a damage metabolite?. FEBS J.

[68] Hudecek O, Benoni R, Reyes-Gutierrez PE, Culka M, Sanderova H, Hubalek M, Rulisek L, Cvacka J, Krasny L, Cahova H (2020). Dinucleoside polyphosphates act as 5'-RNA caps in bacteria. Nat Commun.

[69] Farr SB, Arnosti DN, Chamberlin MJ, Ames BN (1989). An apaH mutation causes AppppA to accumulate and affects motility and catabolite repression in *Escherichia coli*. Proc Natl Acad Sci USA.

[70] Ismail TM, Hart CA, McLennan AG (2003). Regulation of dinucleoside polyphosphate pools by the YgdP and ApaH hydrolases is essential for the ability of *Salmonella enterica* serovar typhimurium to invade cultured mammalian cells. J Biol Chem.

[71] Goerlich O, Foeckler R, Holler E (1982). Mechanism of synthesis of adenosine(5')tetraphospho(5')adenosine (AppppA) by aminoacyl-tRNA synthetases. Eur J Biochem.

[72] Fraga H, Fontes R (2011). Enzymatic synthesis of mono and dinucleoside polyphosphates. Biochim Biophys Acta.

[73] Petersen LM, Tisa LS (2014). Molecular characterization of protease activity in Serratia sp. strain SCBI and its importance in cytotoxicity and virulence. J Bacteriol.

[74] Kinscherf TG, Willis DK (2002). Global regulation by gidA in *Pseudomonas syringae*. J Bacteriol.

[75] Shippy DC, Fadl AA (2015). RNA modification enzymes encoded by the gid operon: implications in biology and virulence of bacteria. Microb Pathog.

[76] Shin JH, Lee HW, Kim SM, Kim J (2009). Proteomic analysis of *Acinetobacter baumannii* in biofilm and planktonic growth mode. J Microbiol.

[77] Zhang W, Zhao Z, Zhang B, Wu XG, Ren ZG, Zhang LQ (2014). Posttranscriptional regulation of 2,4-diacetylphloroglucinol production by GidA and TrmE in *Pseudomonas fluorescens* 2P24. Appl Environ Microbiol.

[78] Li D, Shibata Y, Takeshita T, Yamashita Y (2014). A novel gene involved in the survival of *Streptococcus mutans* under stress conditions. Appl Environ Microbiol.

[79] Yi H, Lee H, Cho KH, Kim HS (2018). Mutations in MetG (methionyl-tRNA synthetase) and TrmD [tRNA (guanine-N1)-methyltransferase] conferring meropenem tolerance in *Burkholderia thailandensis*. J Antimicrob Chemother.

[80] Brauner A, Fridman O, Gefen O, Balaban NQ (2016). Distinguishing between resistance, tolerance and persistence to antibiotic treatment. Nat Rev Microbiol.

[81] Fridman O, Goldberg A, Ronin I, Shoresh N, Balaban NQ (2014). Optimization of lag time underlies antibiotic tolerance in evolved bacterial populations. Nature.

[82] Bolt EL, Jenkins T, Russo VM, Ahmed S, Cavey J, Cass SD (2015). Identification of *Escherichia coli* ygaQ and rpmG as novel mitomycin C resistance factors implicated in DNA repair. Biosci Rep.

[83] Smirnova GV, Oktyabrsky ON (2005). Glutathione in bacteria. Biochemistry.

[84] Jamieson DJ (1998). Oxidative stress responses of the yeast *Saccharomyces cerevisiae*. Yeast.

[85] Van Laar TA, Esani S, Birges TJ, Hazen B, Thomas JM, Rawat M (2018). Pseudomonas aeruginosa gshA mutant is defective in biofilm formation, swarming, and pyocyanin production. mSphere.

[86] Wongsaroj L, Saninjuk K, Romsang A, Duang-Nkern J, Trinachartvanit W, Vattanaviboon P, Mongkolsuk S (2018). Pseudomonas aeruginosa glutathione biosynthesis genes play multiple roles in stress protection, bacterial virulence and biofilm formation. PLoS ONE.

[87] Gomez MJ, Neyfakh AA (2006). Genes involved in intrinsic antibiotic resistance of *Acinetobacter baylyi*. Antimicrob Agents Chemother.

[88] Feinbaum RL, Urbach JM, Liberati NT, Djonovic S, Adonizio A, Carvunis AR, Ausubel FM (2012). Genome-wide identification of *Pseudomonas aeruginosa* virulence-related genes using a *Caenorhabditis elegans* infection model. PLoS Pathog.

[89] Song M, Husain M, Jones-Carson J, Liu L, Henard CA, Vazquez-Torres A (2013). Low-molecular-weight thiol-dependent antioxidant and antinitrosative defences in Salmonella pathogenesis. Mol Microbiol.

[90] Westley J (1981). Thiosulfate: cyanide sulfurtransferase (rhodanese). Methods Enzymol.

[91] Donadio S, Shafiee A, Hutchinson CR (1990). Disruption of a rhodaneselike gene results in cysteine auxotrophy in *Saccharopolyspora erythraea*. J Bacteriol.

[92] Pagani S, Bonomi F, Cerletti P (1984). Enzymic synthesis of the iron-sulfur cluster of spinach ferredoxin. Eur J Biochem.

[93] Bonomi F, Pagani S, Kurtz DM (1985). Enzymic synthesis of the 4Fe-4S clusters of *Clostridium pasteurianum* ferredoxin. Eur J Biochem.

[94] Navidifar T, Amin M, Rashno M (2019). Effects of sub-inhibitory concentrations of meropenem and tigecycline on the expression of genes regulating pili, efflux pumps and virulence factors involved in biofilm formation by *Acinetobacter baumannii*. Infect Drug Resist.

[95] Yang CH, Su PW, Moi SH, Chuang LY (2019). Biofilm formation in *Acinetobacter Baumannii*: genotype-phenotype correlation. Molecules.

[96] Gaddy JA, Tomaras AP, Actis LA (2009). The *Acinetobacter baumannii* 19606 OmpA protein plays a role in biofilm formation on abiotic surfaces and in the interaction of this pathogen with eukaryotic cells. Infect Immun.

[97] Confer AW, Ayalew S (2013). The OmpA family of proteins: roles in bacterial pathogenesis and immunity. Vet Microbiol.

[98] Skerniskyte J, Karazijaite E, Deschamps J, Krasauskas R, Briandet R, Suziedeliene E (2019). The mutation of conservative Asp268 residue in the peptidoglycan-associated domain of the OmpA protein affects multiple *Acinetobacter baumannii* virulence characteristics. Molecules.

[99] Insua JL, Llobet E, Moranta D, Perez-Gutierrez C, Tomas A, Garmendia J, Bengoechea JA (2013). Modeling *Klebsiella pneumoniae* pathogenesis by infection of the wax moth *Galleria mellonella*. Infect Immun.

[100] Justice SS, Hunstad DA, Cegelski L, Hultgren SJ (2008). Morphological plasticity as a bacterial survival strategy. Nat Rev Microbiol.

[101] Robertson GT, Case ED, Dobbs N, Ingle C, Balaban M, Celli J, Norgard MV (2014). FTT0831c/FTL_0325 contributes to *Francisella tularensis* cell division, maintenance of cell shape, and structural integrity. Infect Immun.

[102] Egan AJF (2018). Bacterial outer membrane constriction. Mol Microbiol.

[103] Kim SW, Choi CH, Moon DC, Jin JS, Lee JH, Shin JH, Kim JM, Lee YC, Seol SY, Cho DT, Lee JC (2009). Serum resistance of *Acinetobacter baumannii* through the binding of factor H to outer membrane proteins. FEMS Microbiol Lett.

[104] Choi CH, Lee JS, Lee YC, Park TI, Lee JC (2008). *Acinetobacter baumannii* invades epithelial cells and outer membrane protein A mediates interactions with epithelial cells. BMC Microbiol.

[105] Rumbo-Feal S, Gomez MJ, Gayoso C, Alvarez-Fraga L, Cabral MP, Aransay AM, Rodriguez-Ezpeleta N, Fullaondo A, Valle J, Tomas M, Bou G, Poza M (2013). Whole transcriptome analysis of *Acinetobacter baumannii* assessed by RNA-sequencing reveals different mRNA expression profiles in biofilm compared to planktonic cells. PLoS ONE.

[106] Chang KC, Kuo HY, Tang CY, Chang CW, Lu CW, Liu CC, Lin HR, Chen KH, Liou ML (2014). Transcriptome profiling in imipenem-selected *Acinetobacter baumannii*. BMC Genomics.

[107] Gebhardt MJ, Gallagher LA, Jacobson RK, Usacheva EA, Peterson LR, Zurawski DV, Shuman HA (2015). Joint transcriptional control of virulence and resistance to antibiotic and environmental stress in *Acinetobacter baumannii*. MBio.

[108] Blaschke U, Suwono B, Zafari S, Ebersberger I, Skiebe E, Jeffries CM, Svergun DI, Wilharm G (2018). Recombinant production of A1S_0222 from *Acinetobacter baumannii* ATCC 17978 and confirmation of its DNA-(adenine N6)-methyltransferase activity. Protein Expr Purif.

[109] Roberts RJ, Vincze T, Posfai J, Macelis D (2015). REBASE-a database for DNA restriction and modification: enzymes, genes and genomes. Nucleic Acids Res.

[110] Wion D, Casadesus J (2006). N6-methyl-adenine: an epigenetic signal for DNA-protein interactions. Nat Rev Microbiol.

[111] Adhikari S, Curtis PD (2016). DNA methyltransferases and epigenetic regulation in bacteria. FEMS Microbiol Rev.

[112] Marinus MG, Morris NR (1973). Isolation of deoxyribonucleic acid methylase mutants of *Escherichia coli* K-12. J Bacteriol.

[113] Collier J (2009). Epigenetic regulation of the bacterial cell cycle. Curr Opin Microbiol.

[114] Salzer R, Kern T, Joos F, Averhoff B (2016). The *Thermus thermophilus* comEA/comEC operon is associated with DNA binding and regulation of the DNA translocator and type IV pili. Environ Microbiol.

[115] Rabinovich L, Sigal N, Borovok I, Nir-Paz R, Herskovits AA (2012). Prophage excision activates Listeria competence genes that promote phagosomal escape and virulence. Cell.

[116] Yoshida A, Kuramitsu HK (2002). Multiple *Streptococcus mutans* genes are involved in biofilm formation. Appl Environ Microbiol.

[117] Jacobs AC, Thompson MG, Black CC, Kessler JL, Clark LP, McQueary CN, Gancz HY, Corey BW, Moon JK, Si Y, Owen MT, Hallock JD, Kwak YI, Summers A, Li CZ, Rasko DA, Penwell WF, Honnold CL, Wise MC, Waterman PE, Lesho EP, Stewart RL, Actis LA, Palys TJ, Craft DW, Zurawski DV (2014). AB5075, a highly virulent isolate of *Acinetobacter baumannii*, as a model strain for the evaluation of pathogenesis and antimicrobial treatments. MBio.

[118] Harms K, Lunnan A, Hulter N, Mourier T, Vinner L, Andam CP, Marttinen P, Fridholm H, Hansen AJ, Hanage WP, Nielsen KM, Willerslev E, Johnsen PJ (2016). Substitutions of short heterologous DNA segments of intragenomic or extragenomic origins produce clustered genomic polymorphisms. Proc Natl Acad Sci USA.

[119] Domingues S, Harms K, Fricke WF, Johnsen PJ, da Silva GJ, Nielsen KM (2012). Natural transformation facilitates transfer of transposons, integrons and gene cassettes between bacterial species. PLoS Pathog.

