# Supplementary material for: Novel Genes Required for Surface-Associated Motility in Acinetobacter baumannii
Source: Curr Microbiol. 2021 Mar 5;78(4):1509–28. doi: 10.1007/s00284-021-02407-x (PMC7997844; doi:10.1007/s00284-021-02407-x)
Supplement: Supplementary file 1 — (DOCX 5213 KB) [file 284_2021_2407_MOESM1_ESM.docx]

**Novel genes required for surface-associated motility in**

***Acinetobacter baumannii***

Ulrike Blaschke^*^, Evelyn Skiebe and Gottfried Wilharm^*^

Robert Koch Institute, Project group P2, Burgstr. 37, D-38855 Wernigerode, Germany

* Corresponding addresses: [ulrikeblaschke@googlemail.com](mailto:ulrikeblaschke@googlemail.com); [WilharmG@rki.de](mailto:WilharmG@rki.de)

**Supplementary material**

**
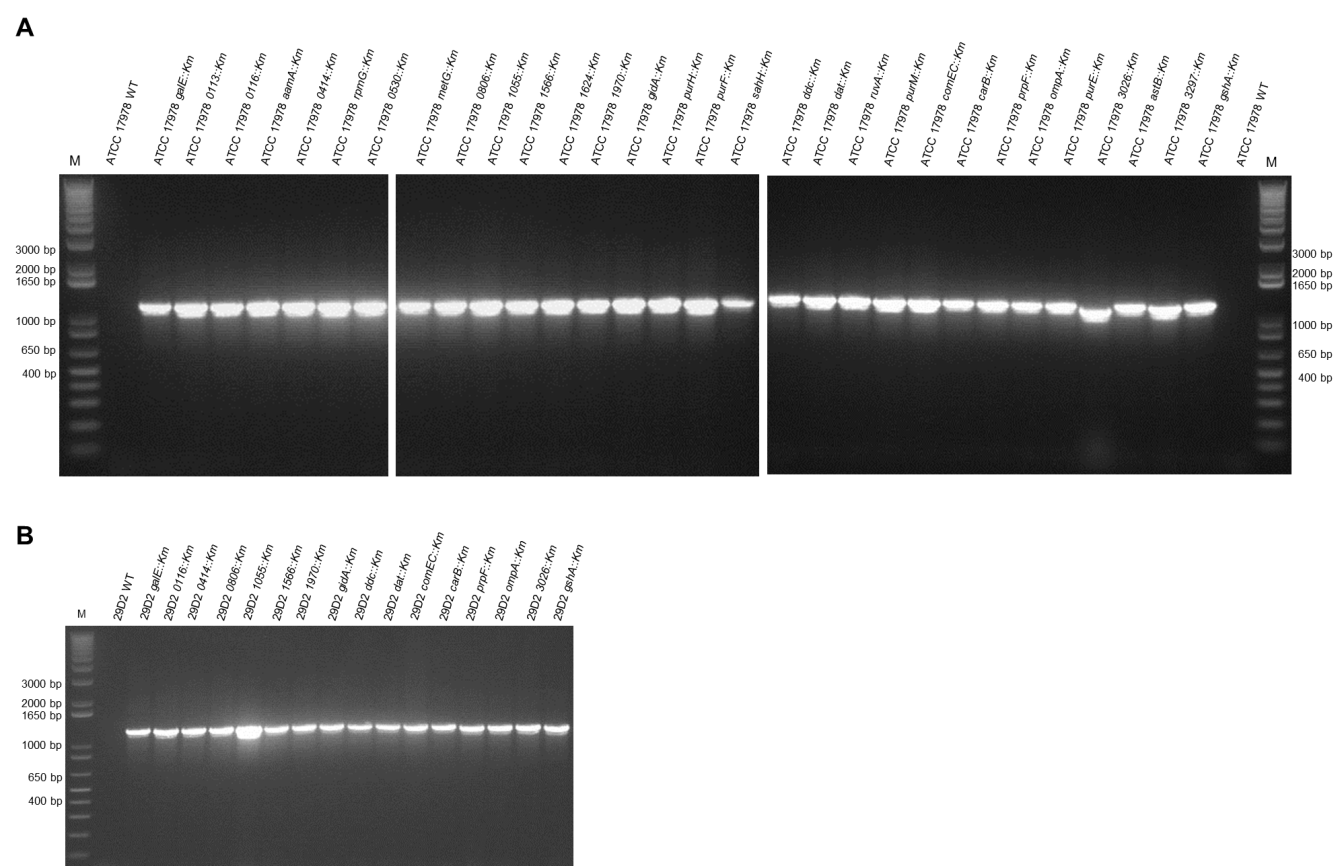
**

**Fig. S1. PCR confirmation of EZ-Tn5™ <KAN-2> transposon insertion in ATCC 17978 (A) and 29D2 (B) mutants.** PCR using the kanamycin cassette primers of the EZ-Tn5™ <KAN-2> insertion kit (Epicentre Biotechnologies). Insertion of the EZ-Tn5™ <KAN-2> transposon results in a 1221 bp PCR product. Both wildtype strains, ATCC 17978 WT (A) and 29D2 WT (B), are used as a negative control.

**
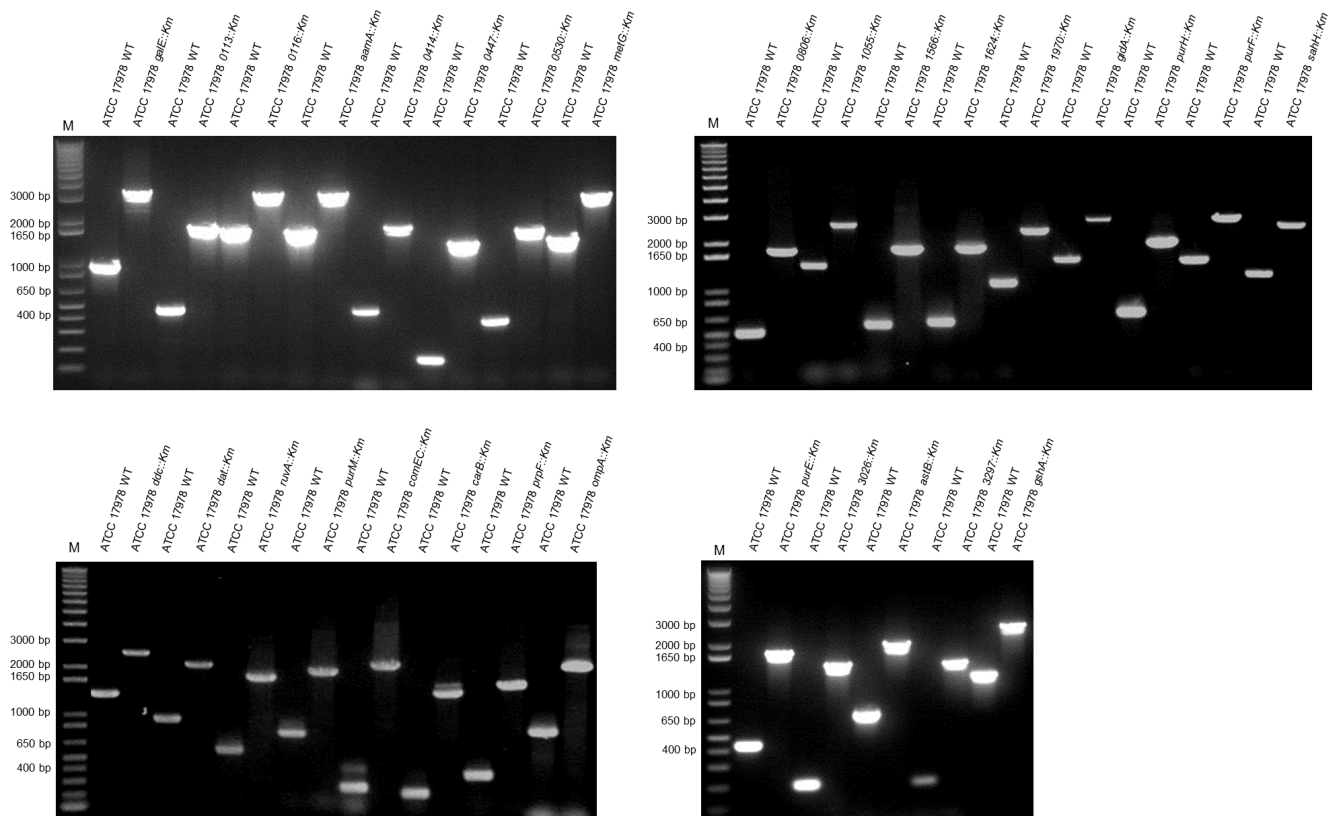
**

**Fig. S2. PCR confirmation of specific gene inactivation in strain ATCC 17978.** For each mutant, specific gene target site primers are used as described in Materials & Methods and Table S2. The expected size of PCR products for each mutant is given in Table S2.

**
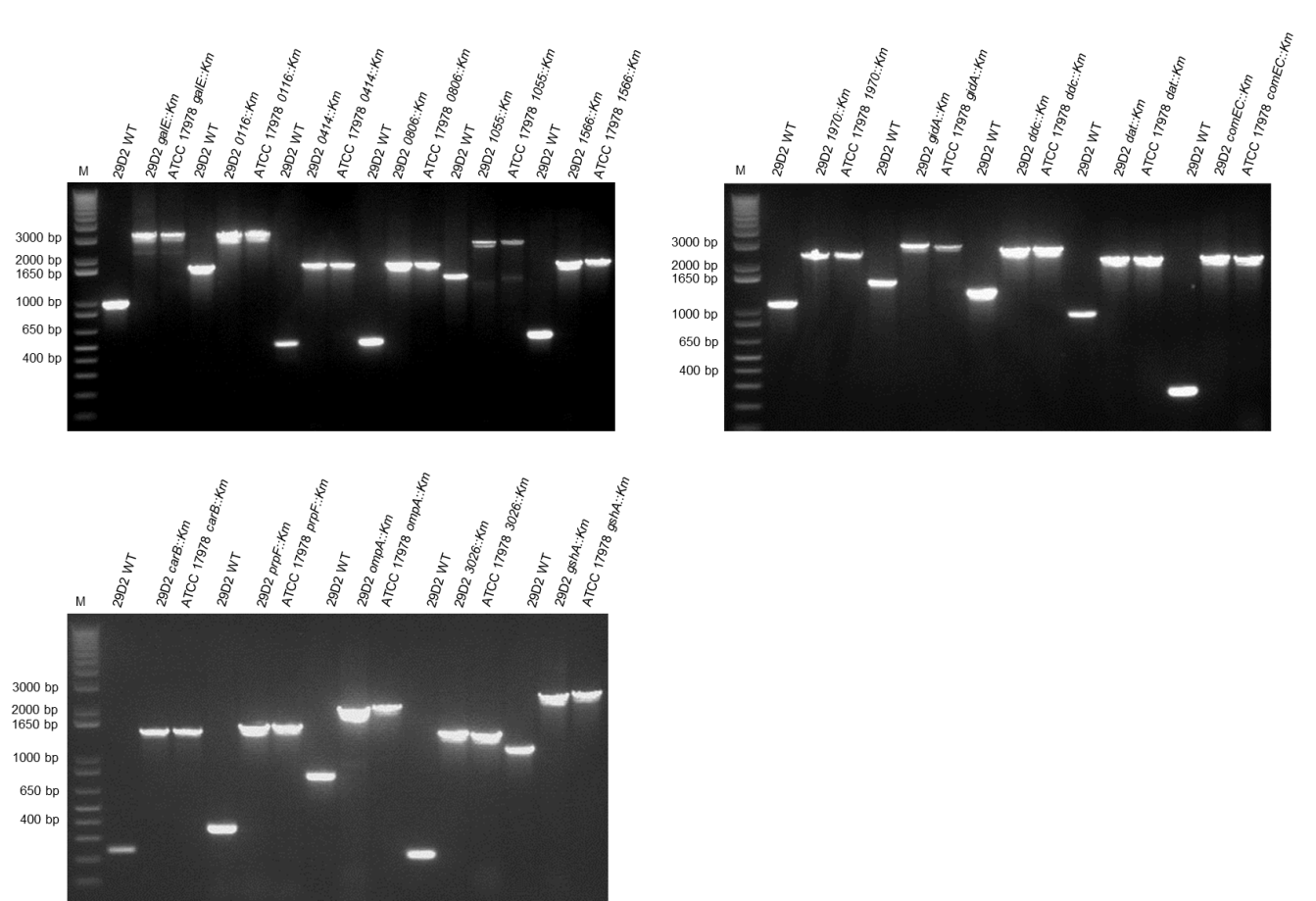
**

**Fig. S3. PCR confirmation of specific gene inactivation in strain 29D2.** Appropriate gene target site primers are used as described in Materials & Methods and Table S2. Defined length of PCR products for each mutant is given in Table S2.

**
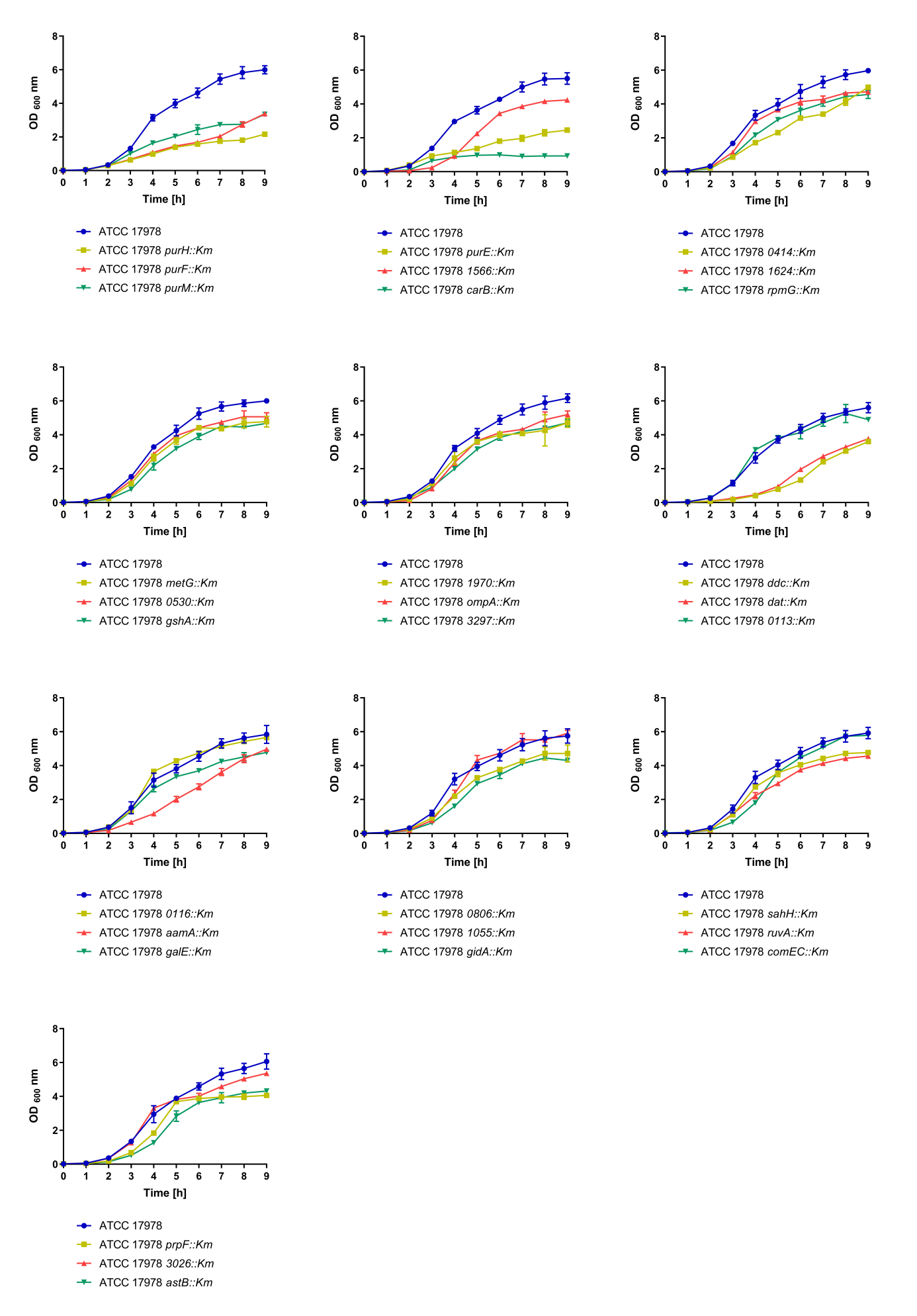
**

**Fig. S4. Growth curves of ATCC 17978 wildtype and mutant strains.** OD-adjusted bacterial cultures were incubated for 9 hours in baffled flasks at 37°C under constant shaking. Every hour cultures were measured at an OD of 600 nm. For each strain data obtained from three independent cultures grown on the same day were averaged and represented by the mean ± SD. The ATCC 17978 wildtype is indicated by a blue line.

**
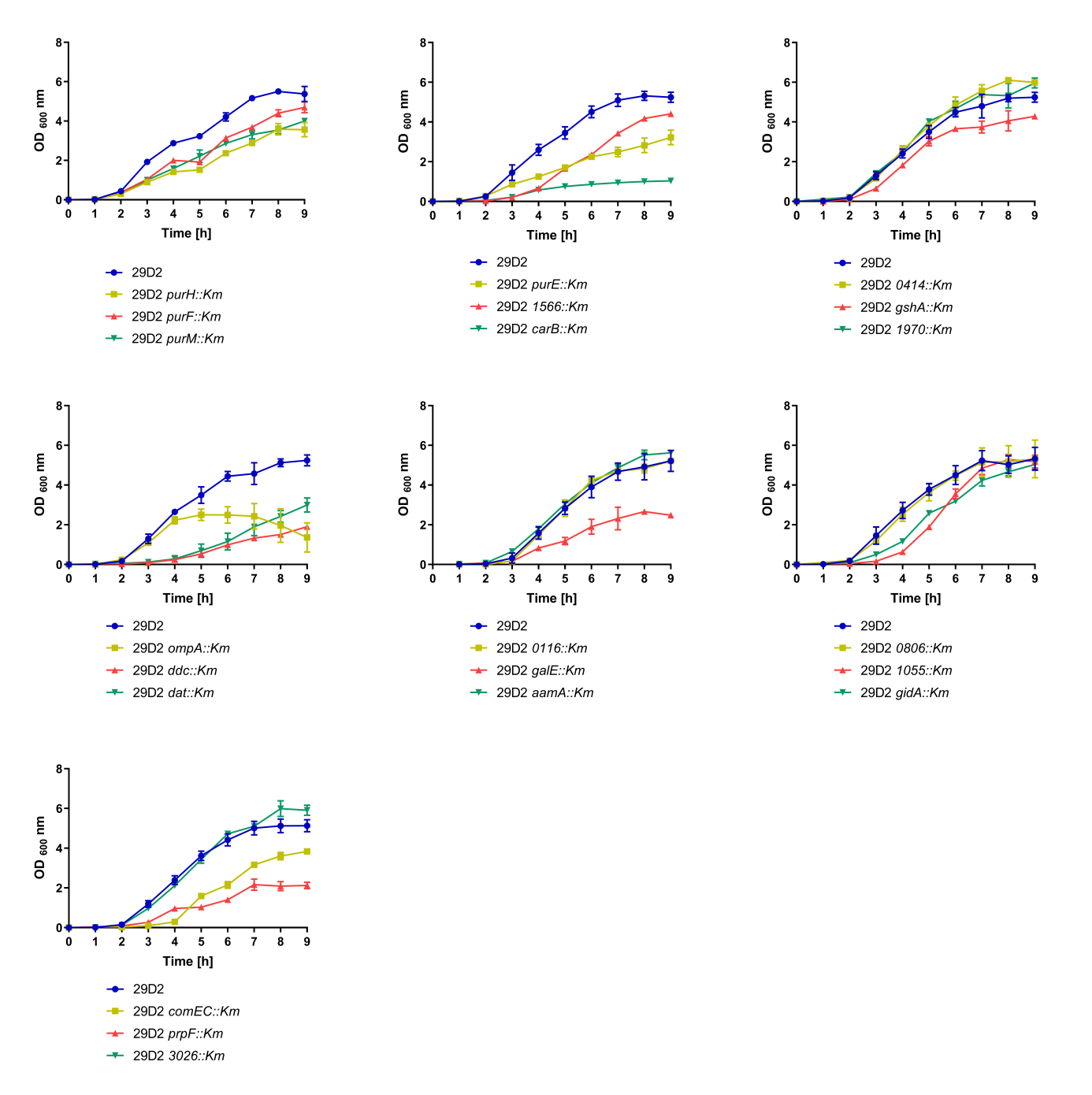
**

**Fig. S5. Growth curves of 29D2 wildtype strain and 29D2 mutants.** OD adjusted overnight cultures were incubated for 9 hours in baffled flasks at 37°C under constant shaking. Every hour cultures were measured at an OD of 600 nm. For each strain data obtained from three independent cultures grown on the same day were averaged and represented by the mean ± SD. The 29D2 wildtype is indicated by a blue line.

**
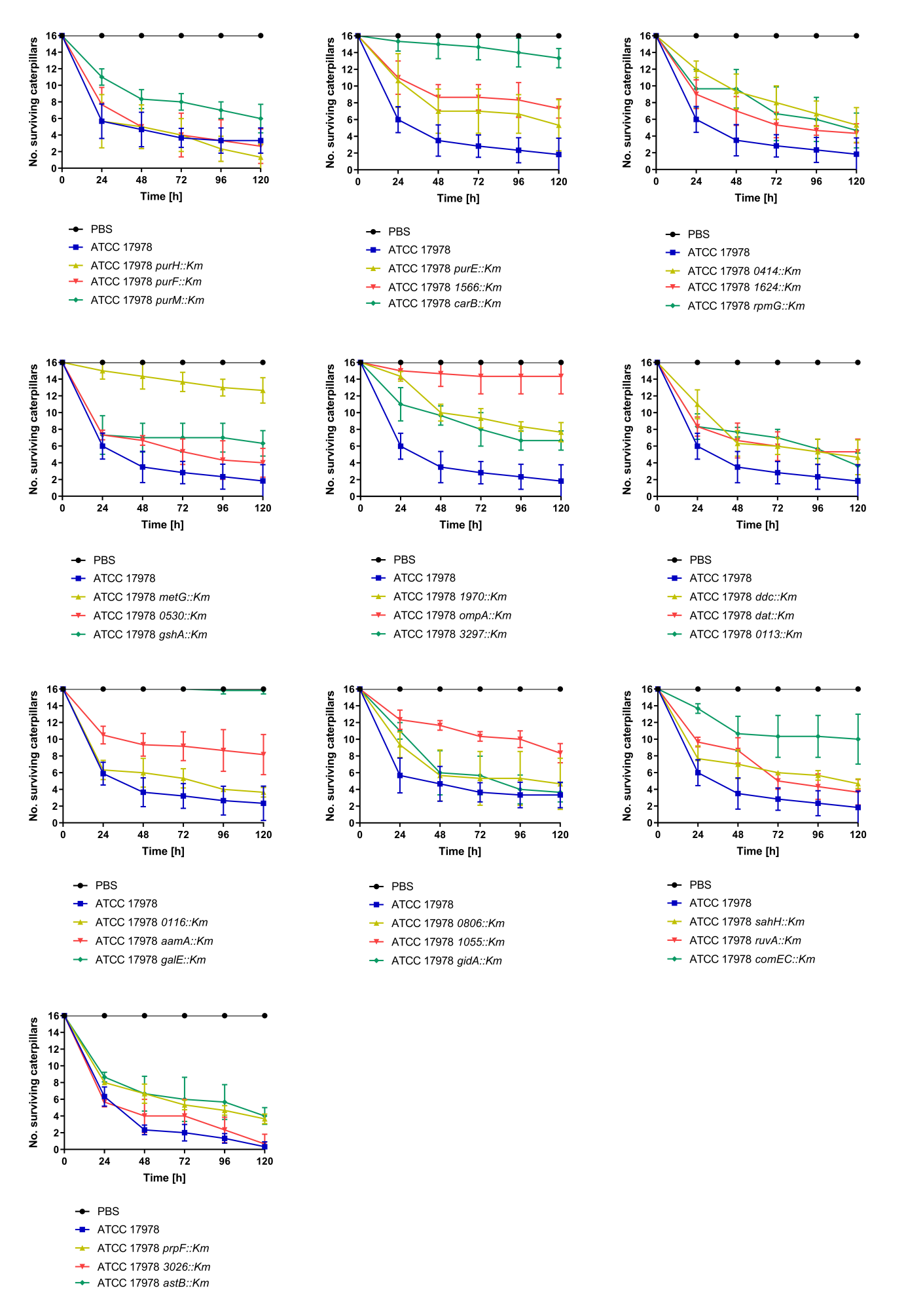
**

**Fig. S6. *Galleria mellonella* caterpillars infected with ATCC 17978 wildtype and mutant strains.** Caterpillars were infected with 3 x 10^5^ CFU of either ATCC 17978 wildtype (blue line) or mutant strains. Sterile PBS was used as a control (black line). Three independent experiments were performed with groups of 16 caterpillars for every bacteria strain and control. Data obtained from three independent experiments were averaged and represented by the mean ± SD. Significant attenuation in caterpillar infection is observed for mutants *carB::Km*, *ompA::Km*, *metG::Km* and *galE::Km* after 5 days post infection.

**
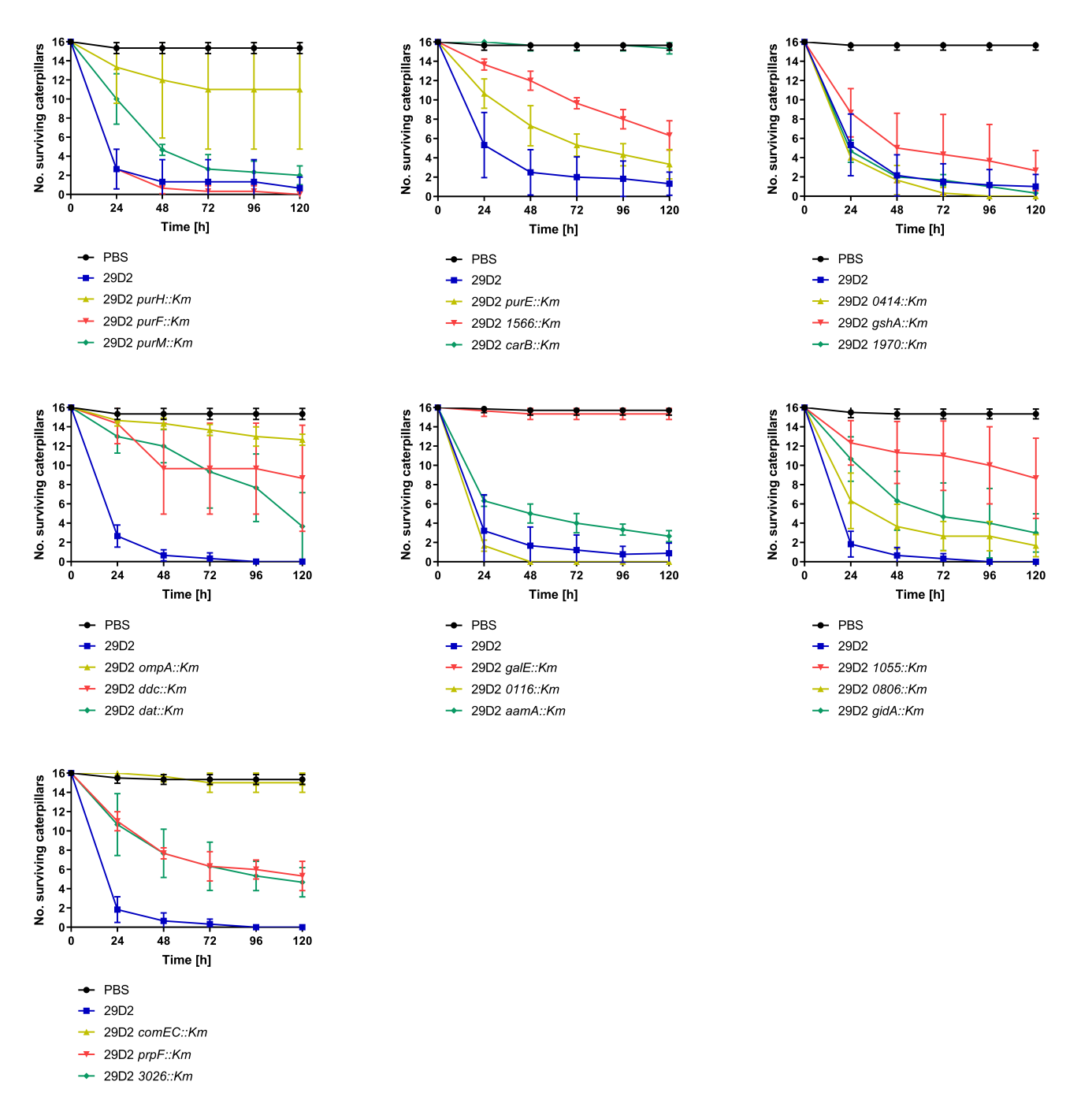
**

**Fig. S7. Infection of *Galleria mellonella* caterpillars with 29D2 wildtype and 29D2 mutant strains.** *G. mellonella* caterpillars were infected with 3 x 10^5^ CFU of 29D2 wildtype (blue line) or mutant strains. As a control sterile PBS was used (black line). Three independent experiments were performed with groups of 16 caterpillars for every bacterial strain and control. Data obtained from three independent experiments were averaged and represented by the mean ± SD. Significant attenuation after 5 days p.i. is observed for mutants *carB::Km*, *ompA::Km*, *galE::Km* and *comEC::Km.*

**
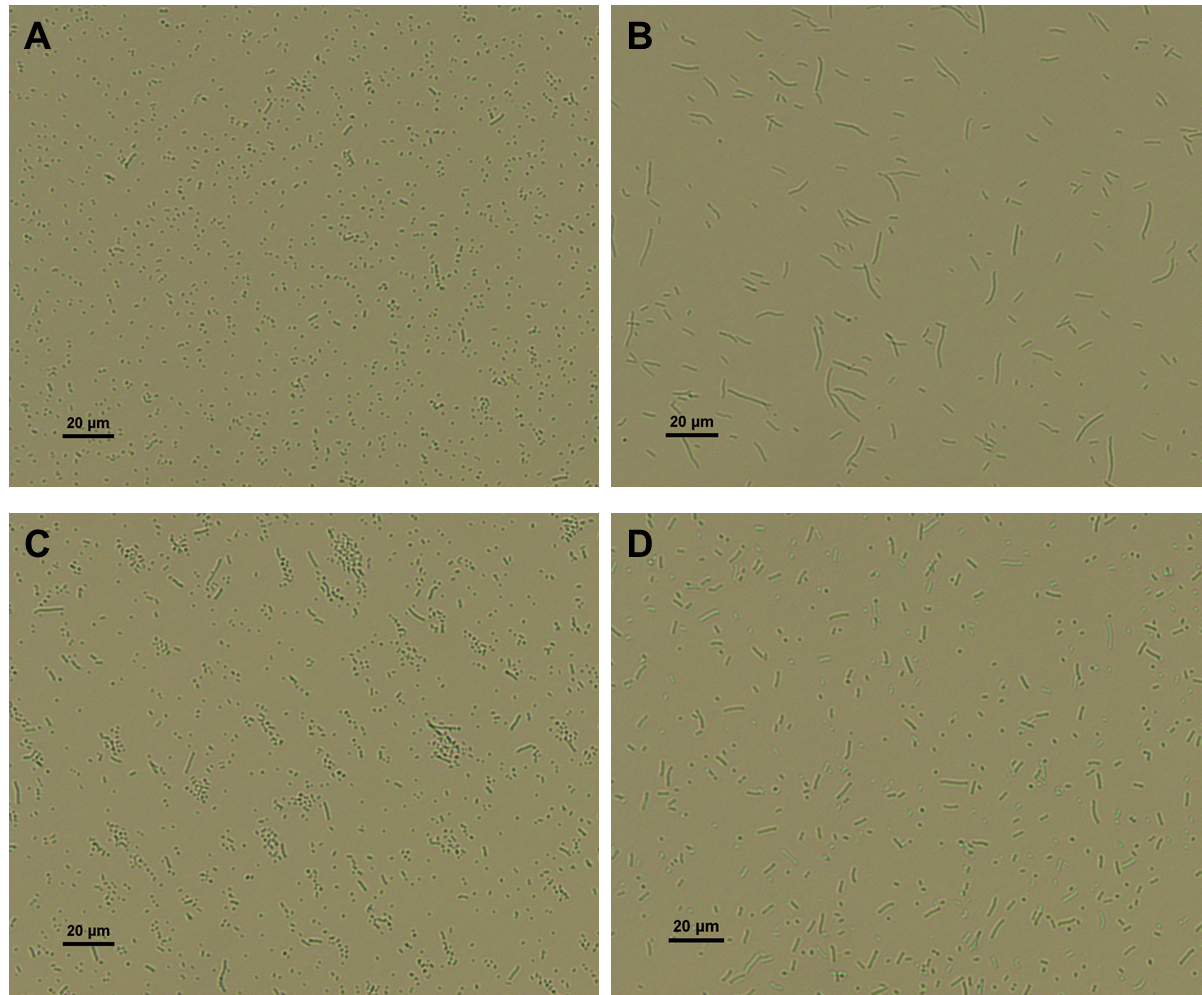
**

**Fig. S8. Bright field microscopy of *ompA::Km* mutants (B,D) and parental strains (A,C).** Bacterial cells were grown for 16 hours at 37°C under constant shaking. One microliter of each bacterial strain was pipetted on a glass slide and analyzed under the bright field microscope (200-fold magnification). The ATCC 17978 wildtype strain **(A)** and 29D2 wildtype strain **(C)** display round to rod-shaped bacterial cells. Both, the ATTC 17978 *ompA::Km* mutant **(B)** and the 29D2 *ompA::Km* mutant **(D)** display an elongated or chain structure phenotype.

**Table S1. Bacterial strains used in this work**

| Bacteria strain | **Genotype** | **Source** |
| --- | --- | --- |
| *A. baumannii* ATCC 17978 | wildtype strain | American Type Culture Collection (ATCC) |
| ATCC 17978 *0065::Km* | A1S_0065::EZ-Tn5™ <KAN-2> (*galE*) | This work |
| ATCC 17978 *0113::Km* | A1S_0113::EZ-Tn5™ <KAN-2> | This work |
| ATCC 17978 *0116::Km* | A1S_0116::EZ-Tn5™ <KAN-2> | This work |
| ATCC 17978 *aamA::Km* | A1S_0222::EZ-Tn5™ <KAN-2> (*aamA*) | [1] |
| ATCC 17978 *0414::Km* | A1S_0414::EZ-Tn5™ <KAN-2> | This work |
| ATCC 17978 *0447::Km* | A1S_0447::EZ-Tn5™ <KAN-2> (*rpmG*) | This work |
| ATCC 17978 *0530::Km* | A1S_0530::EZ-Tn5™ <KAN-2> | This work |
| ATCC 17978 *0778::Km* | A1S_0778::EZ-Tn5™ <KAN-2> (*metG*) | This work |
| ATCC 17978 *0806::Km* | A1S_0806::EZ-Tn5™ <KAN-2> | This work |
| ATCC 17978 *1055::Km* | A1S_1055::EZ-Tn5™ <KAN-2> | This work |
| ATCC 17978 *1566::Km* | A1S_1566::EZ-Tn5™ <KAN-2> | This work |
| ATCC 17978 *1624::Km* | A1S_1624::EZ-Tn5™ <KAN-2> | This work |
| ATCC 17978 *1970::Km* | A1S_1970::EZ-Tn5™ <KAN-2> | This work |
| ATCC 17978 *gidA::Km* | A1S_2182::EZ-Tn5™ <KAN-2> (*gidA*) | This work |
| ATCC 17978 *purH::Km* | A1S_2187::EZ-Tn5™ <KAN-2> (*purH*) | This work |
| ATCC 17978 *purF::Km* | A1S_2251::EZ-Tn5™ <KAN-2> (*purF*) | This work |
| ATCC 17978 *sahH::Km* | A1S_2334::EZ-Tn5™ <KAN-2> (*sahH*) | This work |
| ATCC 17978 *ddc::Km* | A1S_2453::EZ-Tn5™ <KAN-2> (*ddc*) | [2] |
| ATCC 17978 *dat::Km* | A1S_2454::EZ-Tn5™ <KAN-2> (*dat*) | [2] |
| ATCC 17978 *2587::Km* | A1S_2587::EZ-Tn5™ <KAN-2> (*ruvA*) | This work |
| ATCC 17978 *purM::Km* | A1S_2605::EZ-Tn5™ <KAN-2> (*purM*) | This work |
| ATCC 17978 *comEC::Km* | A1S_2610::EZ-Tn5™ <KAN-2> (*comEC*) | [3] |
| ATCC 17978 *2687::Km* | A1S_2687::EZ-Tn5™ <KAN-2> (*carB*) | This work |
| ATCC 17978 *prpF::Km* | A1S_2761::EZ-Tn5™ <KAN-2> (*prpF*) | This work |
| ATCC 17978 *ompA::Km* | A1S_2840::EZ-Tn5™ <KAN-2> (*ompA*) | This work |
| ATCC 17978 *purE::Km* | A1S_2964::EZ-Tn5™ <KAN-2> (*purE*) | This work |
| ATCC 17978 *3026::Km* | A1S_3026::EZ-Tn5™ <KAN-2> | This work |
| ATCC 17978 *astB::Km* | A1S_3129::EZ-Tn5™ <KAN-2> (*astB*) | This work |
| ATCC 17978 *3297::Km* | A1S_3297::EZ-Tn5™ <KAN-2> | This work |
| ATCC 17978 *3366::Km* | A1S_3366::EZ-Tn5™ <KAN-2> (*gshA*) | This work |
| *A. baumannii* 29D2 | wildtype strain | White stork isolate [4] |
| 29D2 *aamA::Km* | A1S_0222::EZ-Tn5™ <KAN-2> (*aamA*) | This work |
| 29D2 *0065::Km* | A1S_0065::EZ-Tn5™ <KAN-2> (*galE*) | This work |
| 29D2 *0116::Km* | A1S_0116::EZ-Tn5™ <KAN-2> | This work |
| 29D2 *0414::Km* | A1S_0414::EZ-Tn5™ <KAN-2> | This work |
| 29D2 *0806::Km* | A1S_0806::EZ-Tn5™ <KAN-2> | This work |
| 29D2 *1055::Km* | A1S_1055::EZ-Tn5™ <KAN-2> | This work |
| 29D2 *1566::Km* | A1S_1566::EZ-Tn5™ <KAN-2> | This work |
| 29D2 *1970::Km* | A1S_1970::EZ-Tn5™ <KAN-2> | This work |
| 29D2 *gidA::Km* | A1S_2182::EZ-Tn5™ <KAN-2> (*gidA*) | This work |
| 29D2 *ddc::Km* | A1S_2453::EZ-Tn5™ <KAN-2> (*ddc*) | This work |
| 29D2 *dat::Km* | A1S_2454::EZ-Tn5™ <KAN-2> (*dat*) | This work |
| 29D2 *comEC::Km* | A1S_2610::EZ-Tn5™ <KAN-2> (*comEC*) | This work |
| 29D2 *2687::Km* | A1S_2687::EZ-Tn5™ <KAN-2> (*carB*) | This work |
| 29D2 *prpF::Km* | A1S_2761::EZ-Tn5™ <KAN-2> (*prpF*) | This work |
| 29D2 *ompA::Km* | A1S_2840::EZ-Tn5™ <KAN-2> (*ompA*) | This work |
| 29D2 *3026::Km* | A1S_3026::EZ-Tn5™ <KAN-2> | This work |
| 29D2 *3366::Km* | A1S_3366::EZ-Tn5™ <KAN-2> (*gshA*) | This work |
| 29D2 *purH::Km* | A1S_2187::EZ-Tn5™ <KAN-2> (*purH*) | This work |
| 29D2 *purF::Km* | A1S_2251::EZ-Tn5™ <KAN-2> (*purF*) | This work |
| 29D2 *purM::Km* | A1S_2605::EZ-Tn5™ <KAN-2> (*purM*) | This work |
| 29D2 *purE::Km* | A1S_2964::EZ-Tn5™ <KAN-2> (*purE*) | This work |

**Table S2. Oligonucleotides used for determination of gene target sites**

| Primer name | **Forward primer (3‘ → 5‘)** | **Reverse primer (5‘ → 3‘)** | | **PCR product in wildtype strain [bp]^a^** | | **Locus tag in ATCC 17978** |
| --- | --- | --- | --- | --- | --- | --- |
| 0065 for/rev | GCAGGTTATATTGGTTCACACAC | | CCATTAGGATTCTGCTTTTGCC | | 980 | A1S_0065 |
| Acyl-CoA-DH for/rev | ATGTCACTTACGTCCAATTTGCG | | CGATTGATCGACCATTTCTCACC | | 488 | A1S_0113 |
| 0116 for/rev | TGACCATAGAACATGCTTTCCTC | | CTTTGCGTTTGACCTTCACAACC | | 1668 | A1S_0116 |
| BamHI-0222 for/rev | GGATCCGGATGAAATGATCAGTTATGTGGC | | GGATCCGTGAGACAGATCCCGTTAGTTGC | | 1649 | A1S_0222 |
| 313/0414 for/rev | TAATTGTTGCACTGGCAACTGTG | | TGATCGACTCAGAAGGTTTCCG | | 479 | A1S_0414 |
| 0447 for/rev | GATTTTAGCTTCTTTGAAAATCACG | | ATGCGTGATAAGATTCGCCTCG | | 150 | A1S_0447 |
| 0530 for/rev | TTTTGGTTTGCTTTTAACGAGAGG | | GGAACGCTGGTTAGAGTTTATGG | | 409 | A1S_0530 |
| 0778 for/rev | CTTTATCGCCAGGTTTTGCACC | | CCTCTTTCAATTGCTAACAAATTGG | | 1432 | A1S_0778 |
| AT_0806 for/rev | GCAACGATTGAACTTGATGATGG | | CTGAATAGCTTGCTCTAGCTCG | | 498 | A1S_0806 |
| MTG_1055 for/rev | ATCCACGTATTGAAGCACAGCG | | TACTGTATAAGTACTACGCTTACC | | 1427 | A1S_1055 |
| 1566 for/rev | GTTTGAAAATGCACACGTTGTACG | | CTTCTACTTGTAAGTTAACATGCG | | 569 | A1S_1566 |
| 1624 for/rev | TTGCATCCACAACTTGCTCAAG | | CGGTGAGGTAAAGGCATAGATC | | 592 | A1S_1624 |
| 1970 for/rev | ATCACTTTTTACACCAAGTACTCC | | TAATGAAATTGCAGTTGAAGCATTC | | 1103 | A1S_1970 |
| GidA_2182 for/rev | GGACGTTTAAGCAAATCAATCGC | | CTATCCTAAAGTTTATGATGTTATCG | | 1542 | A1S_2182 |
| 2187 for/rev | GCCGTGTTAAAACACTACATCC | | TCTAAAGAAACGGCAACACCAC | | 685 | A1S_2187 |
| APT_2251 for/rev | CGGTCAAATCTACTGATGCTGC | | AGTGAACCAAATGTTGTTTGATGC | | 1490 | A1S_2251 |
| 2334 for/rev | AGATTACAAAGTTGCTGACATCTC | | ACTTCTACGCGAATTTTTGCAGC | | 1212 | A1S_2334 |
| 2453 for/rev | CGTAGTTACCAGAAGCAATCGC | | CGTTCAGCAATTTCTTTCCAACC | | 1363 | A1S_2453 |
| AT_2454 for/rev | CACGGAAAGTACCAGTATGACC | | TGAGCGTTACTTCTGTCAACCC | | 957 | A1S_2454 |
| 2587 for/rev | ATGTTTAATTGGCGAAGTGTTTGC | | TACTTCATCATTGATTTAAGTGCAGC | | 591 | A1S_2587 |
| 2605 for/rev | CCGGTTTAAGCTACAAAGATGCG | | AGCTTCATTAATTACAGCTTGAGC | | 800 | A1S_2605 |
| 134/2610 for2 /  2610-comb rev | CAATTAGCAACAGTAGACTTGCG | | CCCACATGTGCTCATTTTTGCC | | 278 | A1S_2610 |
| 172/2687 for/rev | GTTCGTGACAAAAACGACAACTG | | TTCATCTCGATCACAACCATACG | | 242 | A1S_2687 |
| 120/2761 for/rev | CCAGATTCGAACAGTACAGAGGC | | CAGCAACTTACATGCGTGGTGG | | 383 | A1S_2761 |
| OmpA for/rev | TGAGCTGCTGCAGGAGCTGC | | AAGTTAAAGGCGACGTAGACGG | | 826 | A1S_2840 |
| 188/2964 for/rev | CCTGCCCCGGAATATTGTTGC | | ATGGGTTCCCAGTCCGATTGG | | 463 | A1S_2964 |
| Klon1-RNaseT2 for/rev | GCTTGGGCAATTAGAGACAACG | | AAAATACCTGCTAATCTAACCAGC | | 222 | A1S_3026 |
| 25/3129 for/rev | GACTCTTCAGGCACAACAATGG | | CGGAGATGACATTAGTGTTGTGG | | 756 | A1S_3129 |
| 130/3297 for/rev | TTTACTTAACTCCTAAGTTAAGTGTTGG | | AGCCGATAGTTTGTGTATCGTAGC | | 204 | A1S_3297 |
| 354/3366 for/rev | TTAGAAATATGCTGACGTGCCC | | CGTGGAATAGAACGTGAAAGCC | | 1179 | A1S_3366 |

^a^ Based on the insertion of the EZ-Tn5™ <KAN-2> transposon (1221 bp), PCR products in mutant strains are 1221 bp longer than PCR products from wildtype strains

**Table S3. Mean and standard deviation (SD) of ATCC 17978 wildtype/mutants and 29D2 wildtype/mutants surface motility spreading zones**

| **Locus tag** | **Gene name** | **Mean** ± **SD diameter of surface motility spreading zone [mm]^a^** | |
| --- | --- | --- | --- |
|  |  | **ATCC 17978** | **29D2** |
| **Wildtype** |  | 77.5 ± 10.6 | 30 ± 10 |
| **A1S_2187** | *purH* | 10.25 ± 3.5 | 5 ± 1.15 |
| **A1S_2251** | *purF* | 6 ± 0 | 3.75 ± 0.95 |
| **A1S_2605** | *purM* | 3.62 ± 1.10 | 16 ± 1.63 |
| **A1S_2964** | *purE* | 3.5 ± 1.29 | 6.25 ± 1.25 |
| **A1S_1566** |  | 3.75 ± 0.5 | 10 ± 2.94 |
| **A1S_2687** | *carB* | 4.5 ± 2.38 | 7.75 ± 4.19 |
| **A1S_0414** |  | 4.37 ± 1.10 | 12.25 ± 2.62 |
| **A1S_1624** |  | 7.25 ± 0.95 | - |
| **A1S_0447** | *rpmG* | 3.75 ± 1.70 | - |
| **A1S_0778** | *metG* | 2.62 ± 0.47 | - |
| **A1S_0530** |  | 5 ± 1.82 | - |
| **A1S_3366** | *gshA* | 4.875 ± 1.54 | 6.5 ± 1.29 |
| **A1S_1970** |  | 8.75 ± 4.27 | 8.75 ± 3.40 |
| **A1S_2840** | *ompA* | 6.5 ± 4.04 | 10.5 ± 1 |
| **A1S_3297** |  | 11 ± 2.16 | - |
| **A1S_2453** | *ddc* | 4 ± 1.41 | 4.25 ± 1.25 |
| **A1S_2454** | *dat* | 3 ± 1.82 | 7.75 ± 2.06 |
| **A1S_0113** |  | 4.75 ± 0.95 | - |
| **A1S_0116** |  | 5 ± 1.82 | 10 ± 4.89 |
| **A1S_0222** | *aamA* | 4.75 ± 2.75 | 7.75 ± 3.30 |
| **A1S_0065** | *galE* | 4.5 ± 1.29 | 5.5 ± 1.91 |
| **A1S_0806** |  | 1 ± 0.81 | 7 ± 1.15 |
| **A1S_1055** |  | 5 ± 0.81 | 8.25 ± 2.06 |
| **A1S_2182** | *gidA* | 4.25 ± 0.5 | 7.75 ± 0.5 |
| **A1S_2334** | *sahH* | 3.75 ± 1.70 | - |
| **A1S_2587** | *ruvA* | 5 ± 1.41 | - |
| **A1S_2610** | *comEC* | 5.5 ± 2.38 | 6.25 ± 1.70 |
| **A1S_2761** | *prpF* | 4 ± 1.41 | 9.5 ± 0.57 |
| **A1S_3026** |  | 4.87 ± 0.25 | 9 ± 2.58 |
| **A1S_3129** | *astB* | 3 ± 0.40 | - |

^a^ For each strain three independent experiments were performed and represented by the mean ± SD

**Table S4. Mean and standard deviation (SD) of ATCC 17978 wildtype/mutants and 29D2 wildtype/mutants pellicle biofilm measurements**

| **Locus tag** | **Gene name** | **Mean** ± **SD [OD _550 nm_]^a^** | |
| --- | --- | --- | --- |
|  |  | **ATCC 17978** | **29D2** |
| **Wildtype** |  | 8.602 ± 1.519 | 5.560 ± 1.330 |
| **A1S_2187** | *purH* | 11.403 ± 1.540 | 7.266 ± 0.716 |
| **A1S_2251** | *purF* | 6.786 ± 1.065 | 5.98 ± 0.128 |
| **A1S_2605** | *purM* | 10.663 ± 0.831 | 4.793 ± 0.902 |
| **A1S_2964** | *purE* | 11.883 ± 0.387 | 4.71 ± 0.918 |
| **A1S_1566** |  | 1.806 ± 0.969 | 7.303 ± 3.210 |
| **A1S_2687** | *carB* | 0.570 ± 0.195 | 3.166 ± 0.707 |
| **A1S_0414** |  | 0.343 ± 0.211 | 5.156 ± 1.207 |
| **A1S_1624** |  | 7.95 ± 1.063 | - |
| **A1S_0447** | *rpmG* | 9.943 ± 2.089 | - |
| **A1S_0778** | *metG* | 2.259 ± 0.397 | - |
| **A1S_0530** |  | 13.1 ± 1.375 | - |
| **A1S_3366** | *gshA* | 9.573 ± 1.160 | 5.976 ± 0.206 |
| **A1S_1970** |  | 5.633 ± 1.896 | 3.136 ± 0.770 |
| **A1S_2840** | *ompA* | 1.064 ± 0.609 | 3.93 ± 2.170 |
| **A1S_3297** |  | 11.093 ± 1.165 | - |
| **A1S_2453** | *ddc* | 3.213 ± 1.868 | 2.606 ± 0.669 |
| **A1S_2454** | *dat* | 2.308 ± 1.619 | 3.203 ± 0.090 |
| **A1S_0113** |  | 5.726 ± 0.781 | - |
| **A1S_0116** |  | 5.283 ± 1.504 | 4.196 ± 0.342 |
| **A1S_0222** | *aamA* | 4.123 ± 2.237 | 6.748 ± 1.626 |
| **A1S_0065** | *galE* | 14.943 ± 2.844 | 7.723 ± 1.337 |
| **A1S_0806** |  | 15.88 ± 2.104 | 5.963 ± 0.601 |
| **A1S_1055** |  | 11.4 ± 1.018 | 7.126 ± 0.977 |
| **A1S_2182** | *gidA* | 2.556 ± 0.295 | 3.883 ± 0.261 |
| **A1S_2334** | *sahH* | 9.95 ± 0.645 | - |
| **A1S_2587** | *ruvA* | 4.4 ± 1.368 | - |
| **A1S_2610** | *comEC* | 8.813 ± 3.092 | 4.486 ± 0.680 |
| **A1S_2761** | *prpF* | 0.687 ± 0.405 | 6.21 ± 0.425 |
| **A1S_3026** |  | 8.6 ± 0.781 | 3.426 ± .0327 |
| **A1S_3129** | *astB* | 4.066 ± 1.888 | - |

^a^ For each strain three independent experiments were performed and represented by the mean ± SD

**Table S5. Mean and standard deviation (SD) of bacterial growth measurement from ATCC 17978 wildtype/mutants and 29D2 wildtype/mutants**

| **Locus tag** | **Gene name** | **Mean ± SD [OD _600 nm_]^a^** | |
| --- | --- | --- | --- |
|  |  | **ATCC 17978** | **29D2** |
| **Wildtype** |  | 5.87 ± 0.37 | 5.31 ± 0.36 |
| **A1S_2187** | *purH* | 2.16 ± 0.07 | 3.56 ± 0.36 |
| **A1S_2251** | *purF* | 3.38 ± 0.14 | 4.69 ± 0.27 |
| **A1S_2605** | *purM* | 3.35 ± 0.07 | 4.00 ± 0.10 |
| **A1S_2964** | *purE* | 2.45 ± 0.12 | 3.22 ± 0.37 |
| **A1S_1566** |  | 4.24 ± 0.08 | 4.41 ± 0.13 |
| **A1S_2687** | *carB* | 0.92 ± 0.008 | 1.03 ± 0.04 |
| **A1S_0414** |  | 4.99 ± 0.07 | 5.99 ± 0.07 |
| **A1S_1624** |  | 4.72 ± 0.11 | - |
| **A1S_0447** | *rpmG* | 4.56 ± 0.23 | - |
| **A1S_0778** | *metG* | 4.77 ± 0.31 | - |
| **A1S_0530** |  | 5.06 ± 0.24 | - |
| **A1S_3366** | *gshA* | 4.68 ± 0.15 | 4.28 ± 0.02 |
| **A1S_1970** |  | 4.70 ± 0.27 | 5.96 ± 0.24 |
| **A1S_2840** | *ompA* | 5.19 ± 0.21 | 1.36 ± 0.73 |
| **A1S_3297** |  | 4.71 ± 0.20 | - |
| **A1S_2453** | *ddc* | 3.60 ± 0.14 | 1.90 ± 0.13 |
| **A1S_2454** | *dat* | 3.76 ± 0.14 | 2.99 ± 0.35 |
| **A1S_0113** |  | 4.89 ± 0.11 | - |
| **A1S_0116** |  | 5.66 ± 0.07 | 5.21 ± 0.10 |
| **A1S_0222** | *aamA* | 4.97 ± 0.06 | 5.62 ± 0.08 |
| **A1S_0065** | *galE* | 4.77 ± 0.12 | 2.48 ± 0.07 |
| **A1S_0806** |  | 4.71 ± 0.50 | 5.31 ± 0.94 |
| **A1S_1055** |  | 5.91 ± 0.16 | 5.18 ± 0.32 |
| **A1S_2182** | *gidA* | 4.31 ± 0.10 | 5.03 ± 0.24 |
| **A1S_2334** | *sahH* | 4.76 ± 0.05 | - |
| **A1S_2587** | *ruvA* | 4.56 ± 0.04 | - |
| **A1S_2610** | *comEC* | 5.78 ± 0.04 | 3.84 ± 0.09 |
| **A1S_2761** | *prpF* | 4.06 ± 0.11 | 2.12 ± 0.16 |
| **A1S_3026** |  | 5.36 ± 0.08 | 5.91 ± 0.25 |
| **A1S_3129** | *astB* | 4.31 ± 0.04 | - |

^a^ Bacterial cultures were incubated for 9 hours at 37°C under shaking. For each strain, data obtained from three independent cultures grown on the same day were averaged. In this table endpoint measurements after 9 hours of growth are represented by the mean ± SD

**Table S6. List of p-values for every monitored time point after *Galleria mellonella* caterpillar infection comparing ATCC 17978 wildtype and mutant strains**

| **Locus tag** | **Gene name** | **p-values^a^** | | | | |
| --- | --- | --- | --- | --- | --- | --- |
|  |  | **24 hours p.i.** | **48 hours p.i.** | **72 hours p.i.** | **96 hours p.i.** | **120 hours p.i.** |
| **A1S_2187** | *purH* | 0.8335 | 0.3506 | 0.3227 | >0.9999 | 0.7110 |
| **A1S_2251** | *purF* | 0.2125 | 0.3506 | 0.3913 | 0.4700 | 0.5708 |
| **A1S_2605** | *purM* | 0.0016 | 0.0050 | 0.0006 | 0.0020 | 0.0166 |
| **A1S_2964** | *purE* | 0.0185 | 0.0524 | 0.0138 | 0.0106 | 0.0698 |
| **A1S_1566** |  | 0.0041 | 0.0045 | 0.0006 | 0.0015 | 0.0030 |
| **A1S_2687** | *carB* | <0.0001 | <0.0001 | <0.0001 | <0.0001 | <0.0001 |
| **A1S_0414** |  | 0.0005 | 0.0037 | 0.0022 | 0.0048 | 0.0412 |
| **A1S_1624** |  | 0.0331 | 0.0306 | 0.0383 | 0.0398 | 0.0835 |
| **A1S_0447** | *rpmG* | 0.0236 | 0.0034 | 0.0334 | 0.0295 | 0.0829 |
| **A1S_0778** | *metG* | <0.0001 | <0.0001 | <0.0001 | <0.0001 | <0.0001 |
| **A1S_0530** |  | 0.2037 | 0.0273 | 0.0383 | 0.1546 | 0.1478 |
| **A1S_3366** | *gshA* | 0.3295 | 0.0306 | 0.0049 | 0.0041 | 0.0104 |
| **A1S_1970** |  | <0.0001 | 0.0009 | 0.0002 | 0.0003 | 0.0022 |
| **A1S_2840** | *ompA* | <0.0001 | <0.0001 | <0.0001 | <0.0001 | <0.0001 |
| **A1S_3297** |  | 0.0041 | 0.0013 | 0.0022 | 0.0034 | 0.0059 |
| **A1S_2453** | *ddc* | 0.0031 | 0.0590 | 0.0087 | 0.0263 | 0.0829 |
| **A1S_2454** | *dat* | 0.0567 | 0.0537 | 0.0179 | 0.0263 | 0.0306 |
| **A1S_0113** |  | 0.0698 | 0.0081 | 0.0021 | 0.0125 | 0.2000 |
| **A1S_0116** |  | 0.7542 | 0.0950 | 0.0282 | 0.1064 | 0.1643 |
| **A1S_0222** | *aamA* | <0.0001 | <0.0001 | <0.0001 | 0.0006 | 0.0008 |
| **A1S_0065** | *galE* | <0.0001 | <0.0001 | <0.0001 | <0.0001 | <0.0001 |
| **A1S_0806** |  | 0.0185 | 0.2196 | 0.1287 | 0.0876 | 0.1270 |
| **A1S_1055** |  | 0.0005 | 0.0002 | <0.0001 | 0.0001 | 0.0012 |
| **A1S_2182** | *gidA* | 0.0016 | 0.1395 | 0.0474 | 0.1778 | 0.1826 |
| **A1S_2334** | *sahH* | 0.1705 | 0.0306 | 0.0053 | 0.0087 | 0.0474 |
| **A1S_2587** | *ruvA* | 0.0063 | 0.0045 | 0.0433 | 0.1036 | 0.2000 |
| **A1S_2610** | *comEC* | <0.0001 | 0.0012 | 0.0005 | 0.0005 | 0.0015 |
| **A1S_2761** | *prpF* | 0.0676 | 0.0335 | 0.0190 | 0.0398 | 0.1643 |
| **A1S_3026** |  | 0.7363 | 0.7220 | 0.3227 | >0.9999 | 0.3778 |
| **A1S_3129** | *astB* | 0.0264 | 0.0537 | 0.0422 | 0.0270 | 0.1190 |

^a^ Compared to ATCC 17978 WT; unpaired t-test was performed after 24, 48, 72, 96, 120 hours p.i.; p-value ≤ 0.05, *; p-value ≤ 0.01, **; p-value ≤ 0.001, ***; p-value ≤ 0.0001, ****

**Table S7.** **List of p-values for every monitored time point after *Galleria mellonella* caterpillar infection comparing 29D2 wildtype and mutant strains**

| **Locus tag** | **Gene name** | **p-values^a^** | | | | |
| --- | --- | --- | --- | --- | --- | --- |
|  |  | **24 hours p.i.** | **48 hours p.i.** | **72 hours p.i.** | **96 hours p.i.** | **120 hours p.i.** |
| **A1S_2187** | *purH* | 0.0129 | 0.0469 | 0.0657 | 0.0657 | 0.0479 |
| **A1S_2251** | *purF* | >0.9999 | 0.6530 | 0.5072 | 0.5072 | 0.3739 |
| **A1S_2605** | *purM* | 0.0196 | 0.0723 | 0.4512 | 0.5391 | 0.2051 |
| **A1S_2964** | *purE* | 0,0390 | 0,0197 | 0,0403 | 0,0719 | 0.0676 |
| **A1S_1566** |  | 0,0046 | 0,0003 | 0,0005 | 0,0011 | 0,0010 |
| **A1S_2687** | *carB* | 0,0012 | <0.0001 | <0.0001 | <0.0001 | <0.0001 |
| **A1S_0414** |  | 0.5087 | 0.7318 | 0.3398 | 0.2625 | 0.2275 |
| **A1S_3366** | *gshA* | 0.1630 | 0.1730 | 0.1856 | 0.1898 | 0.1705 |
| **A1S_1970** |  | 0.7442 | 0.8998 | 0.8878 | 0.8667 | 0.4248 |
| **A1S_2840** | *ompA* | <0,0001 | <0.0001 | <0.0001 | <0.0001 | <0.0001 |
| **A1S_2453** | *ddc* | 0,0011 | 0,0307 | 0,0274 | 0,0240 | 0,0527 |
| **A1S_2454** | *dat* | 0,0010 | 0,0004 | 0,0152 | 0,0194 | 0,1448 |
| **A1S_0116** |  | 0,5020 | 0,1795 | 0,2192 | 0,1486 | 0,1877 |
| **A1S_0222** | *aamA* | 0,1938 | 0,0190 | 0,0176 | 0,0007 | 0,0213 |
| **A1S_0065** | *galE* | 0,0002 | <0.0001 | <0.0001 | <0.0001 | <0.0001 |
| **A1S_0806** |  | 0,0125 | 0,0199 | 0,0092 | 0,0024 | 0,0066 |
| **A1S_1055** |  | <0,0001 | <0,0001 | 0,0001 | 0,0003 | 0,0009 |
| **A1S_2182** | *gidA* | 0,0001 | 0,0027 | 0,0155 | 0,0219 | 0,0054 |
| **A1S_2610** | *comEC* | <0,0001 | <0.0001 | <0.0001 | <0.0001 | <0.0001 |
| **A1S_2761** | *prpF* | <0,0001 | <0,0001 | <0,0001 | <0,0001 | <0,0001 |
| **A1S_3026** |  | 0,0005 | 0,0003 | 0,0005 | <0,0001 | <0,0001 |

^a^ Compared to 29D2 WT; unpaired t-test was performed after 24, 48, 72, 96, 120 hours p.i.; p-value ≤ 0.05, *; p-value ≤ 0.01, **; p-value ≤ 0.001, ***; p-value ≤ 0.0001, ****

**Table S8. Links between genes identified in this study and the literature**

| Locus tag in ATCC 17978 | **Annotation/gene name** | | **Known relationship in other bacteria** | |
| --- | --- | --- | --- | --- |
| *Purine/pyrimidine/folate biosynthesis* | |  | |  |
| A1S_2187 | *purH* | | biofilm formation in *Bacillus cereus* [5]; virulence in *B. anthracis* [6]; K^+^ -dependent colony spreading in *Bacillus subtilis* [7]; *Enterococcus faecium* growth in human serum [8]; defects in rifampicin persistence in *S. aureus* [9] | |
| A1S_2251 | *purF* | | virulence in *A. baumannii* [10]; K^+^ -dependent colony spreading in *Bacillus subtilis* [7]; virulence in *Pasteurella multocida* [11]; virulence of *Burkholderia cenocepacia* in *G. mellonella*, *C. elegans*, *D. melanogaster* infection [12]; defects in rifampicin persistence in *S. aureus* [9] | |
| A1S_2605 | *purM* | | virulence in *A. baumannii* [10]; K^+^ -dependent colony spreading in *Bacillus subtilis* [7]; defects in rifampicin persistence in *S. aureus* [9]; pellicles in *A. baumannii* ATCC 17978 [13] | |
| A1S_2964 | *purE* | | virulence in *S. pneumoniae* [14] and *A. baumannii* [10]; motility (*purK*) in *A. nosocomialis* [15]; pellicles in *A. baumannii* ATCC 17978 (PurB, PurD) [13] | |
| A1S_1566 |  | | - | |
| A1S_2687 | *carB* | | swimming motility and biofilm formation in *Xanthomonas citri subsp. citri* [16]; virulence in *A. baumannii* [10]; growth of *E. coli* in human serum [17] | |
| *Alarmones/ stress metabolite* | |  | |  |
| A1S_0414 |  | | - | |
| A1S_1624 |  | | motility in *E. coli* [18]; pellicles in *A. baumannii* ATCC 17978 [13]; biofilm formation in *Pseudomonas fluorescens* [19]; virulence in *Salmonella enterica* [20]; antibiotic susceptibility in *E. coli*, *A. baumannii* and *P. aeruginosa* [21]; antibiotic tolerance [22] | |
| *RNA modification/regulation* | |  | |  |
| A1S_0447 | *rpmG* | | mitomycin C resistance in *E. coli* [23] | |
| A1S_0778 | *metG* | | virulence in *A. baumannii* [10]; antibiotic tolerance in *Burkholderia thailandensis* [24] and *E. coli* [25,26]; pellicles in *A. baumannii* ATCC 17978 [13] | |
| A1S_2182 | *gidA* | | review *gid* operon, virulence, motility, biofilm formation, antibiotic resistance, bacterial growth [27]; swarming motility, pellicle biofilm in *Bacillus cereus* [28]; swarming motility in *Serratia* species SCBI [29] and *Pseudomonas syringae* [30]; proteomic analysis in *A. baumannii* [31]; biofilm formation in *Pseudomonas fluorescens* [32] and *Streptococcus mutans* [33] | |
| *Oxidative stress* | |  | |  |
| A1S_0530 |  | | virulence in *Salmonella Typhimurium* [34]; thioredoxin involved in *A. baumannii* virulence [35]; general oxidative stress response genes involved in pellicles in *A. baumannii* ATCC 17978 [13] | |
| A1S_3366 | *gshA* | | swarming and swimming motility in *P. aeruginosa*, decrease in biofilm formation [36]; swimming and twitching motility in  *P. aeruginosa*, increase in biofilm formation [37]; sensitivity to metronidazole and ciprofloxacin in *A. baylyi* [38];  *P. aeruginosa gshA* mutant attenuated in *C. elegans* infection [39]; *Salmonella gshA* mutant attenuated in murine model [40] | |
| *Outer membrane proteins* | |  | |  |
| A1S_1970 |  | | - | |
| A1S_2840 | *ompA* | | *A. nosocomialis* surface-associated motility [15]; biofilm formation [41-43]; bacterial pathogenicity – Review [44]; virulence [45-47]; *A. baumannii* virulence in *C. elegans* [48]; *K. pneumoniae* virulence in *G. mellonella* [49]; antibiotic resistance [50]; pellicles in *A. baumannii* ATCC 17978 [13] | |
| A1S_3297 |  | | general outer membrane proteins involved in pellicles in *A. baumannii* ATCC 17978 [13] | |
| *1,3-diaminopropane biosynthesis* | |  | |  |
| A1S_2453 | *ddc* | | surface-associated motility and virulence in *A. baumannii* [2] | |
| A1S_2454 | *dat* | |  |  |
| *Lipopeptide synthesis/ export* | |  | |  |
| A1S_0113 |  | | temperature dependent antibiotic resistance and surface motility in *A. baumannii* [51]; surface-associated motility in *A. baumannii* and  *A. nosocomialis* [15,52]; pellicle biofilm formation in *A. baumannii* [52]; biofilm formation on abiotic surfaces in *A. baumannii* [53,54]; pellicles in *A. baumannii* ATCC 17978 [13]; imipenem-selected *A. baumannii* [55] | |
| A1S_0116 |  | |  |  |
| *DNA modification/repair/uptake* | | |  | |
| A1S_0222 | *aamA* | | protein purification of *A. baumannii* AamA [1]; review of phenotypes caused by *dam* mutants or *dam* overexpression [56] | |
| A1S_2334 | *sahH* | | biofilm formation [57] | |
| A1S_2587 | *ruvA* | | - | |
| A1S_2610 | *comEC* | | surface motility, twitching motility, virulence in *A. baumannii* [3]; twitching motility in *Thermus thermophiles* [58]; virulence and growth in *L. monocytogenes* [59]; biofilm formation [60] | |
| *Others* | |  | |  |
| A1S_0065 | *galE* | | virulence in *A. baumannii* [10], *Bacillus anthracis* [61], *Streptococcus iniae* [62], *Leptosphaeria maculans* [63]; biofilm formation in *Sinorhizobium meliloti* [64], *Vibrio cholerae* [65], *Bacillus subtilis* [66], *Thermus thermophiles* [67], *Haemophilus parasuis* [68], *Porphyromonas gingivalis* [69], *A. baumannii* [31]; antibiotic resistance/susceptibility in *Porphyromonas gingivalis* [69], *Salmonella typhimurium* [70], *Salmonella typhi* [71]; *A. baumannii* biofilm (*galU*, *galM*) [72]; surface motility of *A. nosocomialis* (*rmlB*) [15] | |
| A1S_0806 |  | | survival, growth and virulence of mycobacteria *bioA* [73-76] | |
| A1S_1055 |  | | lytic transglycosylase (A1S_3027) in *A. nosocomialis* motility [15] | |
| A1S_2761 | *prpF* | | pellicles in *A. baumannii* ATCC 17978 [13] | |
| A1S_3026 |  | | reviews of T2 Family Ribonucleases [77,78]; abiotic surface colonization in *A. baumannii* [79]; colistin resistance in *A. baumannii* [80]; neighbouring gene (A1S_3027) in *A. nosocomialis* motility [15] | |
| A1S_3129 | *astB* | | virulence in *A. baumannii* [10]; pellicles in *A. baumannii* ATCC 17978 [13] | |

**References**

1. Blaschke U, Suwono B, Zafari S, Ebersberger I, Skiebe E, Jeffries CM, Svergun DI, Wilharm G (2018) Recombinant production of A1S_0222 from Acinetobacter baumannii ATCC 17978 and confirmation of its DNA-(adenine N6)-methyltransferase activity. Protein Expr Purif 151:78-85. doi:10.1016/j.pep.2018.06.009

2. Skiebe E, de Berardinis V, Morczinek P, Kerrinnes T, Faber F, Lepka D, Hammer B, Zimmermann O, Ziesing S, Wichelhaus TA, Hunfeld KP, Borgmann S, Grobner S, Higgins PG, Seifert H, Busse HJ, Witte W, Pfeifer Y, Wilharm G (2012) Surface-associated motility, a common trait of clinical isolates of Acinetobacter baumannii, depends on 1,3-diaminopropane. Int J Med Microbiol 302 (3):117-128. doi:10.1016/j.ijmm.2012.03.003

3. Wilharm G, Piesker J, Laue M, Skiebe E (2013) DNA uptake by the nosocomial pathogen Acinetobacter baumannii occurs during movement along wet surfaces. J Bacteriol 195 (18):4146-4153. doi:10.1128/JB.00754-13

4. Wilharm G, Skiebe E, Higgins PG, Poppel MT, Blaschke U, Leser S, Heider C, Heindorf M, Brauner P, Jackel U, Bohland K, Cuny C, Lopinska A, Kaminski P, Kasprzak M, Bochenski M, Ciebiera O, Tobolka M, Zolnierowicz KM, Siekiera J, Seifert H, Gagne S, Salcedo SP, Kaatz M, Layer F, Bender JK, Fuchs S, Semmler T, Pfeifer Y, Jerzak L (2017) Relatedness of wildlife and livestock avian isolates of the nosocomial pathogen Acinetobacter baumannii to lineages spread in hospitals worldwide. Environ Microbiol 19 (10):4349-4364. doi:10.1111/1462-2920.13931

5. Vilain S, Pretorius JM, Theron J, Brozel VS (2009) DNA as an adhesin: Bacillus cereus requires extracellular DNA to form biofilms. Appl Environ Microbiol 75 (9):2861-2868. doi:10.1128/AEM.01317-08

6. Jenkins A, Cote C, Twenhafel N, Merkel T, Bozue J, Welkos S (2011) Role of purine biosynthesis in Bacillus anthracis pathogenesis and virulence. Infect Immun 79 (1):153-166. doi:10.1128/IAI.00925-10

7. Kinsinger RF, Kearns DB, Hale M, Fall R (2005) Genetic requirements for potassium ion-dependent colony spreading in Bacillus subtilis. J Bacteriol 187 (24):8462-8469. doi:10.1128/JB.187.24.8462-8469.2005

8. Zhang X, de Maat V, Guzman Prieto AM, Prajsnar TK, Bayjanov JR, de Been M, Rogers MRC, Bonten MJM, Mesnage S, Willems RJL, van Schaik W (2017) RNA-seq and Tn-seq reveal fitness determinants of vancomycin-resistant Enterococcus faecium during growth in human serum. BMC Genomics 18 (1):893. doi:10.1186/s12864-017-4299-9

9. Yee R, Cui P, Shi W, Feng J, Zhang Y (2015) Genetic Screen Reveals the Role of Purine Metabolism in Staphylococcus aureus Persistence to Rifampicin. Antibiotics (Basel) 4 (4):627-642. doi:10.3390/antibiotics4040627

10. Wang N, Ozer EA, Mandel MJ, Hauser AR (2014) Genome-wide identification of Acinetobacter baumannii genes necessary for persistence in the lung. MBio 5 (3):e01163-01114. doi:10.1128/mBio.01163-14

11. Fuller TE, Kennedy MJ, Lowery DE (2000) Identification of Pasteurella multocida virulence genes in a septicemic mouse model using signature-tagged mutagenesis. Microb Pathog 29 (1):25-38. doi:10.1006/mpat.2000.0365

12. Schwager S, Agnoli K, Kothe M, Feldmann F, Givskov M, Carlier A, Eberl L (2013) Identification of Burkholderia cenocepacia strain H111 virulence factors using nonmammalian infection hosts. Infect Immun 81 (1):143-153. doi:10.1128/IAI.00768-12

13. Kentache T, Ben Abdelkrim A, Jouenne T, De E, Hardouin J (2017) Global Dynamic Proteome Study of a Pellicle-forming Acinetobacter baumannii Strain. Mol Cell Proteomics 16 (1):100-112. doi:10.1074/mcp.M116.061044

14. Polissi A, Pontiggia A, Feger G, Altieri M, Mottl H, Ferrari L, Simon D (1998) Large-scale identification of virulence genes from Streptococcus pneumoniae. Infect Immun 66 (12):5620-5629

15. Clemmer KM, Bonomo RA, Rather PN (2011) Genetic analysis of surface motility in Acinetobacter baumannii. Microbiology 157 (Pt 9):2534-2544. doi:10.1099/mic.0.049791-0

16. Zhuo T, Rou W, Song X, Guo J, Fan X, Kamau GG, Zou H (2015) Molecular study on the carAB operon reveals that carB gene is required for swimming and biofilm formation in Xanthomonas citri subsp. citri. BMC Microbiol 15:225. doi:10.1186/s12866-015-0555-9

17. Samant S, Lee H, Ghassemi M, Chen J, Cook JL, Mankin AS, Neyfakh AA (2008) Nucleotide biosynthesis is critical for growth of bacteria in human blood. PLoS Pathog 4 (2):e37. doi:10.1371/journal.ppat.0040037

18. Farr SB, Arnosti DN, Chamberlin MJ, Ames BN (1989) An apaH mutation causes AppppA to accumulate and affects motility and catabolite repression in Escherichia coli. Proc Natl Acad Sci U S A 86 (13):5010-5014. doi:10.1073/pnas.86.13.5010

19. Monds RD, Newell PD, Wagner JC, Schwartzman JA, Lu W, Rabinowitz JD, O'Toole GA (2010) Di-adenosine tetraphosphate (Ap4A) metabolism impacts biofilm formation by Pseudomonas fluorescens via modulation of c-di-GMP-dependent pathways. J Bacteriol 192 (12):3011-3023. doi:10.1128/JB.01571-09

20. Ismail TM, Hart CA, McLennan AG (2003) Regulation of dinucleoside polyphosphate pools by the YgdP and ApaH hydrolases is essential for the ability of Salmonella enterica serovar typhimurium to invade cultured mammalian cells. J Biol Chem 278 (35):32602-32607. doi:10.1074/jbc.M305994200

21. Ji X, Zou J, Peng H, Stolle AS, Xie R, Zhang H, Peng B, Mekalanos JJ, Zheng J (2019) Alarmone Ap4A is elevated by aminoglycoside antibiotics and enhances their bactericidal activity. Proc Natl Acad Sci U S A 116 (19):9578-9585. doi:10.1073/pnas.1822026116

22. Hansen S, Lewis K, Vulic M (2008) Role of global regulators and nucleotide metabolism in antibiotic tolerance in Escherichia coli. Antimicrob Agents Chemother 52 (8):2718-2726. doi:10.1128/AAC.00144-08

23. Bolt EL, Jenkins T, Russo VM, Ahmed S, Cavey J, Cass SD (2015) Identification of Escherichia coli ygaQ and rpmG as novel mitomycin C resistance factors implicated in DNA repair. Biosci Rep 36 (1):e00290. doi:10.1042/BSR20150249

24. Yi H, Lee H, Cho KH, Kim HS (2018) Mutations in MetG (methionyl-tRNA synthetase) and TrmD [tRNA (guanine-N1)-methyltransferase] conferring meropenem tolerance in Burkholderia thailandensis. J Antimicrob Chemother 73 (2):332-338. doi:10.1093/jac/dkx378

25. Brauner A, Fridman O, Gefen O, Balaban NQ (2016) Distinguishing between resistance, tolerance and persistence to antibiotic treatment. Nat Rev Microbiol 14 (5):320-330. doi:10.1038/nrmicro.2016.34

26. Fridman O, Goldberg A, Ronin I, Shoresh N, Balaban NQ (2014) Optimization of lag time underlies antibiotic tolerance in evolved bacterial populations. Nature 513 (7518):418-421. doi:10.1038/nature13469

27. Shippy DC, Fadl AA (2015) RNA modification enzymes encoded by the gid operon: Implications in biology and virulence of bacteria. Microb Pathog 89:100-107. doi:10.1016/j.micpath.2015.09.008

28. Okshevsky M, Louw MG, Lamela EO, Nilsson M, Tolker-Nielsen T, Meyer RL (2018) A transposon mutant library of Bacillus cereus ATCC 10987 reveals novel genes required for biofilm formation and implicates motility as an important factor for pellicle-biofilm formation. Microbiologyopen 7 (2):e00552. doi:10.1002/mbo3.552

29. Petersen LM, Tisa LS (2014) Molecular characterization of protease activity in Serratia sp. strain SCBI and its importance in cytotoxicity and virulence. J Bacteriol 196 (22):3923-3936. doi:10.1128/JB.01908-14

30. Kinscherf TG, Willis DK (2002) Global regulation by gidA in Pseudomonas syringae. J Bacteriol 184 (8):2281-2286. doi:10.1128/jb.184.8.2281-2286.2002

31. Shin JH, Lee HW, Kim SM, Kim J (2009) Proteomic analysis of Acinetobacter baumannii in biofilm and planktonic growth mode. J Microbiol 47 (6):728-735. doi:10.1007/s12275-009-0158-y

32. Zhang W, Zhao Z, Zhang B, Wu XG, Ren ZG, Zhang LQ (2014) Posttranscriptional regulation of 2,4-diacetylphloroglucinol production by GidA and TrmE in Pseudomonas fluorescens 2P24. Appl Environ Microbiol 80 (13):3972-3981. doi:10.1128/AEM.00455-14

33. Li D, Shibata Y, Takeshita T, Yamashita Y (2014) A novel gene involved in the survival of Streptococcus mutans under stress conditions. Appl Environ Microbiol 80 (1):97-103. doi:10.1128/AEM.02549-13

34. Wallrodt I, Jelsbak L, Thorndahl L, Thomsen LE, Lemire S, Olsen JE (2013) The putative thiosulfate sulfurtransferases PspE and GlpE contribute to virulence of Salmonella Typhimurium in the mouse model of systemic disease. PLoS One 8 (8):e70829. doi:10.1371/journal.pone.0070829

35. May HC, Yu JJ, Zhang H, Wang Y, Cap AP, Chambers JP, Guentzel MN, Arulanandam BP (2019) Thioredoxin-A is a virulence factor and mediator of the type IV pilus system in Acinetobacter baumannii. PLoS One 14 (7):e0218505. doi:10.1371/journal.pone.0218505

36. Van Laar TA, Esani S, Birges TJ, Hazen B, Thomas JM, Rawat M (2018) Pseudomonas aeruginosa gshA Mutant Is Defective in Biofilm Formation, Swarming, and Pyocyanin Production. mSphere 3 (2). doi:10.1128/mSphere.00155-18

37. Wongsaroj L, Saninjuk K, Romsang A, Duang-Nkern J, Trinachartvanit W, Vattanaviboon P, Mongkolsuk S (2018) Pseudomonas aeruginosa glutathione biosynthesis genes play multiple roles in stress protection, bacterial virulence and biofilm formation. PLoS One 13 (10):e0205815. doi:10.1371/journal.pone.0205815

38. Gomez MJ, Neyfakh AA (2006) Genes involved in intrinsic antibiotic resistance of Acinetobacter baylyi. Antimicrob Agents Chemother 50 (11):3562-3567. doi:10.1128/AAC.00579-06

39. Feinbaum RL, Urbach JM, Liberati NT, Djonovic S, Adonizio A, Carvunis AR, Ausubel FM (2012) Genome-wide identification of Pseudomonas aeruginosa virulence-related genes using a Caenorhabditis elegans infection model. PLoS Pathog 8 (7):e1002813. doi:10.1371/journal.ppat.1002813

40. Song M, Husain M, Jones-Carson J, Liu L, Henard CA, Vazquez-Torres A (2013) Low-molecular-weight thiol-dependent antioxidant and antinitrosative defences in Salmonella pathogenesis. Mol Microbiol 87 (3):609-622. doi:10.1111/mmi.12119

41. Navidifar T, Amin M, Rashno M (2019) Effects of sub-inhibitory concentrations of meropenem and tigecycline on the expression of genes regulating pili, efflux pumps and virulence factors involved in biofilm formation by Acinetobacter baumannii. Infect Drug Resist 12:1099-1111. doi:10.2147/IDR.S199993

42. Yang CH, Su PW, Moi SH, Chuang LY (2019) Biofilm Formation in Acinetobacter Baumannii: Genotype-Phenotype Correlation. Molecules 24 (10). doi:10.3390/molecules24101849

43. Gaddy JA, Tomaras AP, Actis LA (2009) The Acinetobacter baumannii 19606 OmpA protein plays a role in biofilm formation on abiotic surfaces and in the interaction of this pathogen with eukaryotic cells. Infect Immun 77 (8):3150-3160. doi:10.1128/IAI.00096-09

44. Confer AW, Ayalew S (2013) The OmpA family of proteins: roles in bacterial pathogenesis and immunity. Vet Microbiol 163 (3-4):207-222. doi:10.1016/j.vetmic.2012.08.019

45. Antunes LC, Imperi F, Carattoli A, Visca P (2011) Deciphering the multifactorial nature of Acinetobacter baumannii pathogenicity. PLoS One 6 (8):e22674. doi:10.1371/journal.pone.0022674

46. Kim SW, Choi CH, Moon DC, Jin JS, Lee JH, Shin JH, Kim JM, Lee YC, Seol SY, Cho DT, Lee JC (2009) Serum resistance of Acinetobacter baumannii through the binding of factor H to outer membrane proteins. FEMS Microbiol Lett 301 (2):224-231. doi:10.1111/j.1574-6968.2009.01820.x

47. Choi CH, Lee JS, Lee YC, Park TI, Lee JC (2008) Acinetobacter baumannii invades epithelial cells and outer membrane protein A mediates interactions with epithelial cells. BMC Microbiol 8:216. doi:10.1186/1471-2180-8-216

48. Skerniskyte J, Karazijaite E, Deschamps J, Krasauskas R, Briandet R, Suziedeliene E (2019) The Mutation of Conservative Asp268 Residue in the Peptidoglycan-Associated Domain of the OmpA Protein Affects Multiple Acinetobacter baumannii Virulence Characteristics. Molecules 24 (10). doi:10.3390/molecules24101972

49. Insua JL, Llobet E, Moranta D, Perez-Gutierrez C, Tomas A, Garmendia J, Bengoechea JA (2013) Modeling Klebsiella pneumoniae pathogenesis by infection of the wax moth Galleria mellonella. Infect Immun 81 (10):3552-3565. doi:10.1128/IAI.00391-13

50. Kwon HI, Kim S, Oh MH, Na SH, Kim YJ, Jeon YH, Lee JC (2017) Outer membrane protein A contributes to antimicrobial resistance of Acinetobacter baumannii through the OmpA-like domain. J Antimicrob Chemother 72 (11):3012-3015. doi:10.1093/jac/dkx257

51. De Silva PM, Chong P, Fernando DM, Westmacott G, Kumar A (2018) Effect of Incubation Temperature on Antibiotic Resistance and Virulence Factors of Acinetobacter baumannii ATCC 17978. Antimicrob Agents Chemother 62 (1). doi:10.1128/AAC.01514-17

52. Giles SK, Stroeher UH, Eijkelkamp BA, Brown MH (2015) Identification of genes essential for pellicle formation in Acinetobacter baumannii. BMC Microbiol 15:116. doi:10.1186/s12866-015-0440-6

53. Rumbo-Feal S, Gomez MJ, Gayoso C, Alvarez-Fraga L, Cabral MP, Aransay AM, Rodriguez-Ezpeleta N, Fullaondo A, Valle J, Tomas M, Bou G, Poza M (2013) Whole transcriptome analysis of Acinetobacter baumannii assessed by RNA-sequencing reveals different mRNA expression profiles in biofilm compared to planktonic cells. PLoS One 8 (8):e72968. doi:10.1371/journal.pone.0072968

54. Rumbo-Feal S, Perez A, Ramelot TA, Alvarez-Fraga L, Vallejo JA, Beceiro A, Ohneck EJ, Arivett BA, Merino M, Fiester SE, Kennedy MA, Actis LA, Bou G, Poza M (2017) Contribution of the A. baumannii A1S_0114 Gene to the Interaction with Eukaryotic Cells and Virulence. Front Cell Infect Microbiol 7:108. doi:10.3389/fcimb.2017.00108

55. Chang KC, Kuo HY, Tang CY, Chang CW, Lu CW, Liu CC, Lin HR, Chen KH, Liou ML (2014) Transcriptome profiling in imipenem-selected Acinetobacter baumannii. BMC Genomics 15:815. doi:10.1186/1471-2164-15-815

56. Collier J (2009) Epigenetic regulation of the bacterial cell cycle. Curr Opin Microbiol 12 (6):722-729. doi:10.1016/j.mib.2009.08.005

57. Redanz S, Standar K, Podbielski A, Kreikemeyer B (2012) Heterologous expression of sahH reveals that biofilm formation is autoinducer-2-independent in Streptococcus sanguinis but is associated with an intact activated methionine cycle. J Biol Chem 287 (43):36111-36122. doi:10.1074/jbc.M112.379230

58. Salzer R, Kern T, Joos F, Averhoff B (2016) The Thermus thermophilus comEA/comEC operon is associated with DNA binding and regulation of the DNA translocator and type IV pili. Environ Microbiol 18 (1):65-74. doi:10.1111/1462-2920.12820

59. Rabinovich L, Sigal N, Borovok I, Nir-Paz R, Herskovits AA (2012) Prophage excision activates Listeria competence genes that promote phagosomal escape and virulence. Cell 150 (4):792-802. doi:10.1016/j.cell.2012.06.036

60. Yoshida A, Kuramitsu HK (2002) Multiple Streptococcus mutans Genes Are Involved in Biofilm Formation. Appl Environ Microbiol 68 (12):6283-6291. doi:10.1128/aem.68.12.6283-6291.2002

61. Chateau A, Lunderberg JM, Oh SY, Abshire T, Friedlander A, Quinn CP, Missiakas DM, Schneewind O (2018) Galactosylation of the Secondary Cell Wall Polysaccharide of Bacillus anthracis and Its Contribution to Anthrax Pathogenesis. J Bacteriol 200 (5). doi:10.1128/JB.00562-17

62. Zeng Y, He Y, Wang KY, Wang J, Zeng YK, Chen YX, Chen D, Geng Y, OuYang P (2016) cpsJ gene of Streptococcus iniae is involved in capsular polysaccharide synthesis and virulence. Antonie Van Leeuwenhoek 109 (11):1483-1492. doi:10.1007/s10482-016-0750-1

63. Remy E, Meyer M, Blaise F, Simon UK, Kuhn D, Balesdent MH, Rouxel T (2009) A key enzyme of the Leloir pathway is involved in pathogenicity of Leptosphaeria maculans toward oilseed rape. Mol Plant Microbe Interact 22 (6):725-736. doi:10.1094/MPMI-22-6-0725

64. Schaper S, Wendt H, Bamberger J, Sieber V, Schmid J, Becker A (2019) A Bifunctional UDP-Sugar 4-Epimerase Supports Biosynthesis of Multiple Cell Surface Polysaccharides in Sinorhizobium meliloti. J Bacteriol 201 (10). doi:10.1128/JB.00801-18

65. Nesper J, Lauriano CM, Klose KE, Kapfhammer D, Kraiss A, Reidl J (2001) Characterization of Vibrio cholerae O1 El tor galU and galE mutants: influence on lipopolysaccharide structure, colonization, and biofilm formation. Infect Immun 69 (1):435-445. doi:10.1128/IAI.69.1.435-445.2001

66. Chai Y, Beauregard PB, Vlamakis H, Losick R, Kolter R (2012) Galactose metabolism plays a crucial role in biofilm formation by Bacillus subtilis. MBio 3 (4):e00184-00112. doi:10.1128/mBio.00184-12

67. Niou YK, Wu WL, Lin LC, Yu MS, Shu HY, Yang HH, Lin GH (2009) Role of galE on biofilm formation by Thermus spp. Biochem Biophys Res Commun 390 (2):313-318. doi:10.1016/j.bbrc.2009.09.120

68. Zou Y, Feng S, Xu C, Zhang B, Zhou S, Zhang L, He X, Li J, Yang Z, Liao M (2013) The role of galU and galE of Haemophilus parasuis SC096 in serum resistance and biofilm formation. Vet Microbiol 162 (1):278-284. doi:10.1016/j.vetmic.2012.08.006

69. Nakao R, Senpuku H, Watanabe H (2006) Porphyromonas gingivalis galE is involved in lipopolysaccharide O-antigen synthesis and biofilm formation. Infect Immun 74 (11):6145-6153. doi:10.1128/IAI.00261-06

70. Anton DN (1995) Resistance to mecillinam produced by the co-operative action of mutations affecting lipopolysaccharide, spoT, and cya or crp genes of Salmonella typhimurium. Mol Microbiol 16 (3):587-595. doi:10.1111/j.1365-2958.1995.tb02421.x

71. Hickman FW, Rhoden DL, Esaias AO, Baron LS, Brenner DJ, Farmer JJ, 3rd (1982) Evaluation of two Salmonella typhi strains with reduced virulence for use in teaching and proficiency testing. J Clin Microbiol 15 (6):1085-1091

72. Cabral MP, Soares NC, Aranda J, Parreira JR, Rumbo C, Poza M, Valle J, Calamia V, Lasa I, Bou G (2011) Proteomic and functional analyses reveal a unique lifestyle for Acinetobacter baumannii biofilms and a key role for histidine metabolism. J Proteome Res 10 (8):3399-3417. doi:10.1021/pr101299j

73. Woong Park S, Klotzsche M, Wilson DJ, Boshoff HI, Eoh H, Manjunatha U, Blumenthal A, Rhee K, Barry CE, 3rd, Aldrich CC, Ehrt S, Schnappinger D (2011) Evaluating the sensitivity of Mycobacterium tuberculosis to biotin deprivation using regulated gene expression. PLoS Pathog 7 (9):e1002264. doi:10.1371/journal.ppat.1002264

74. Sassetti CM, Boyd DH, Rubin EJ (2003) Genes required for mycobacterial growth defined by high density mutagenesis. Mol Microbiol 48 (1):77-84. doi:10.1046/j.1365-2958.2003.03425.x

75. Keer J, Smeulders MJ, Gray KM, Williams HD (2000) Mutants of Mycobacterium smegmatis impaired in stationary-phase survival. Microbiology 146 ( Pt 9):2209-2217. doi:10.1099/00221287-146-9-2209

76. Kar R, Nangpal P, Mathur S, Singh S, Tyagi AK (2017) bioA mutant of Mycobacterium tuberculosis shows severe growth defect and imparts protection against tuberculosis in guinea pigs. PLoS One 12 (6):e0179513. doi:10.1371/journal.pone.0179513

77. Deshpande RA, Shankar V (2002) Ribonucleases from T2 family. Crit Rev Microbiol 28 (2):79-122. doi:10.1080/1040-840291046704

78. Luhtala N, Parker R (2010) T2 Family ribonucleases: ancient enzymes with diverse roles. Trends Biochem Sci 35 (5):253-259. doi:10.1016/j.tibs.2010.02.002

79. Jacobs AC, Blanchard CE, Catherman SC, Dunman PM, Murata Y (2014) An ribonuclease T2 family protein modulates Acinetobacter baumannii abiotic surface colonization. PLoS One 9 (1):e85729. doi:10.1371/journal.pone.0085729

80. Mu X, Wang N, Li X, Shi K, Zhou Z, Yu Y, Hua X (2016) The Effect of Colistin Resistance-Associated Mutations on the Fitness of Acinetobacter baumannii. Front Microbiol 7:1715. doi:10.3389/fmicb.2016.01715

81. Lees-Miller RG, Iwashkiw JA, Scott NE, Seper A, Vinogradov E, Schild S, Feldman MF (2013) A common pathway for O-linked protein-glycosylation and synthesis of capsule in Acinetobacter baumannii. Mol Microbiol 89 (5):816-830. doi:10.1111/mmi.12300

82. Russo TA, Luke NR, Beanan JM, Olson R, Sauberan SL, MacDonald U, Schultz LW, Umland TC, Campagnari AA (2010) The K1 capsular polysaccharide of Acinetobacter baumannii strain 307-0294 is a major virulence factor. Infect Immun 78 (9):3993-4000. doi:10.1128/IAI.00366-10

83. Carlson-Banning KM, Chou A, Liu Z, Hamill RJ, Song Y, Zechiedrich L (2013) Toward repurposing ciclopirox as an antibiotic against drug-resistant Acinetobacter baumannii, Escherichia coli, and Klebsiella pneumoniae. PLoS One 8 (7):e69646. doi:10.1371/journal.pone.0069646

84. Shirai H, Mizuguchi K (2003) Prediction of the structure and function of AstA and AstB, the first two enzymes of the arginine succinyltransferase pathway of arginine catabolism. FEBS Lett 555 (3):505-510. doi:10.1016/s0014-5793(03)01314-0
